# Supplementary figures and images for: DECODE enables high-throughput mapping of antibody epitopes at single amino acid resolution
Source: PLoS Biol. 2025 Jan 23;23(1):e3002707. doi: 10.1371/journal.pbio.3002707 (PMC11756784; doi:10.1371/journal.pbio.3002707)

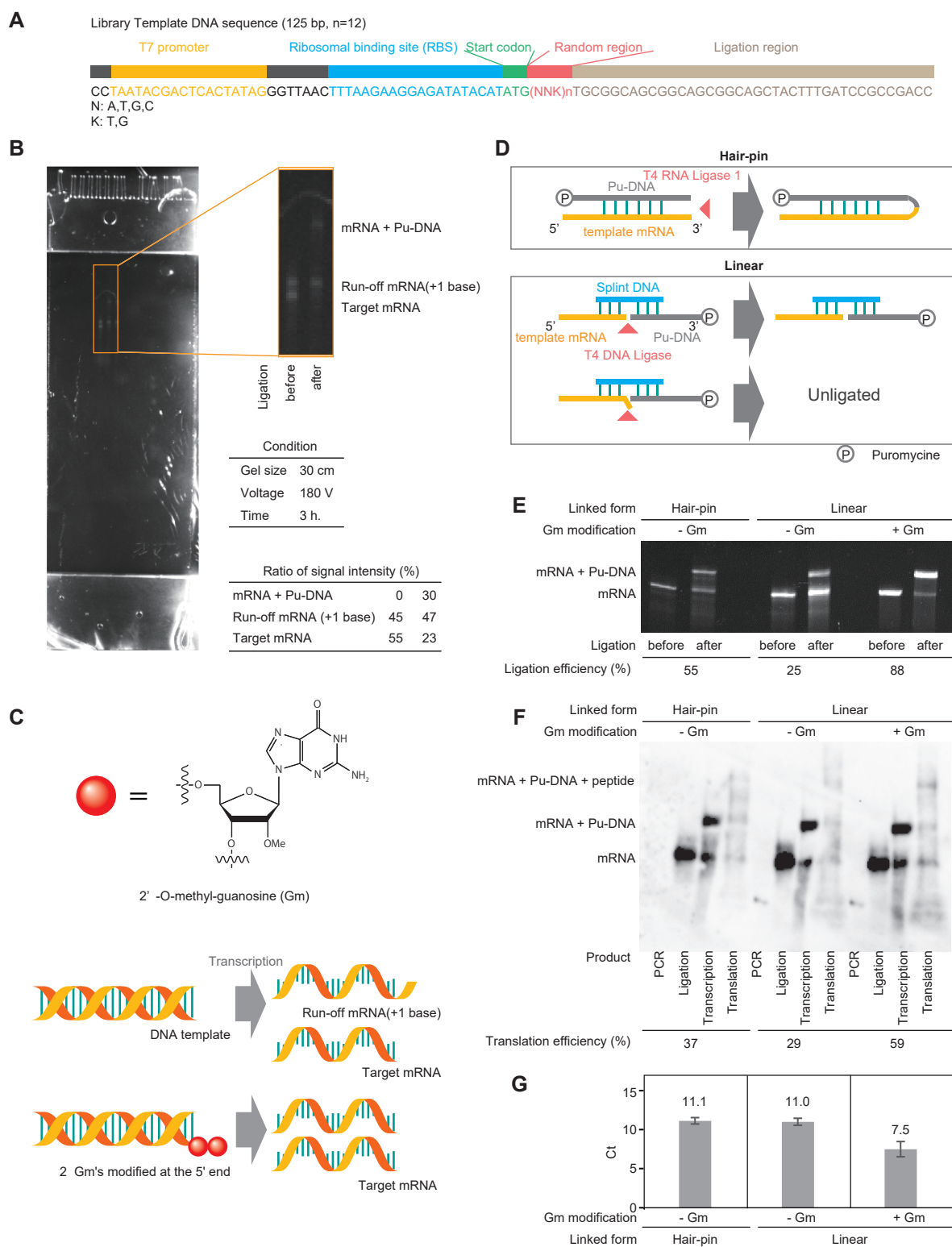

S1 Fig

Supplement: S1 Fig — (A) Template DNA design of DECODE selection. Yellow, blue, green, pink, and gray indicate a T7 promoter, a Shine–Dalgarno sequence, a start codon, a random region, and a linker region, respectively. N and K indicate a mixed nucleotide of A, T, G, C and T, G. NNK is repeated 12 times. (B) Detailed analysis of ligation between Pu-DNA and transcribed mRNA without Gm modified DNA template. Samples are separated in single-nucleotide resolution with a large urea PAGE (10% AA and 6M Urea, 30 cm in length). (C) Schematic illustration of mRNA preparation using 2′-O-methylguanosine (Gm) modified templated DNA. Red spheres indicate Gm. The antisense strand of the 5′ end of the template DNA was modified by 2 Gms to reduce the run-off activity of T7 RNA polymerase. (D) Two kinds of commonly used ligation forms between RNA and Pu-DNA in mRNA display methods are illustrated. The circle included “P” is Puromycin. Linear form ligation is suppressed to a low efficiency by run-off products. (E) Ligation efficiencies of hairpin and linear form using urea PAGE (10% AA and 6 M Urea). (F) Translation efficiencies of hairpin and linear forms using northern blotting (e-PAGEL gradient gel (10% to 20%), nylon membrane (Hybond N+)) and calculated by ImageJ. (G) Quantification of the recovered peptide-cDNA complex using anti-Flag antibody magnetic beads (M2 beads). The recovered complexes used were quantified by qPCR as a threshold cycle (Ct). Data are shown as means ± STD (n = 2). The data underlying for panels G shown in the figure can be found in S2 Data or https://doi.org/10.5281/zenodo.14286317. (PDF) [file pbio.3002707.s001.pdf]

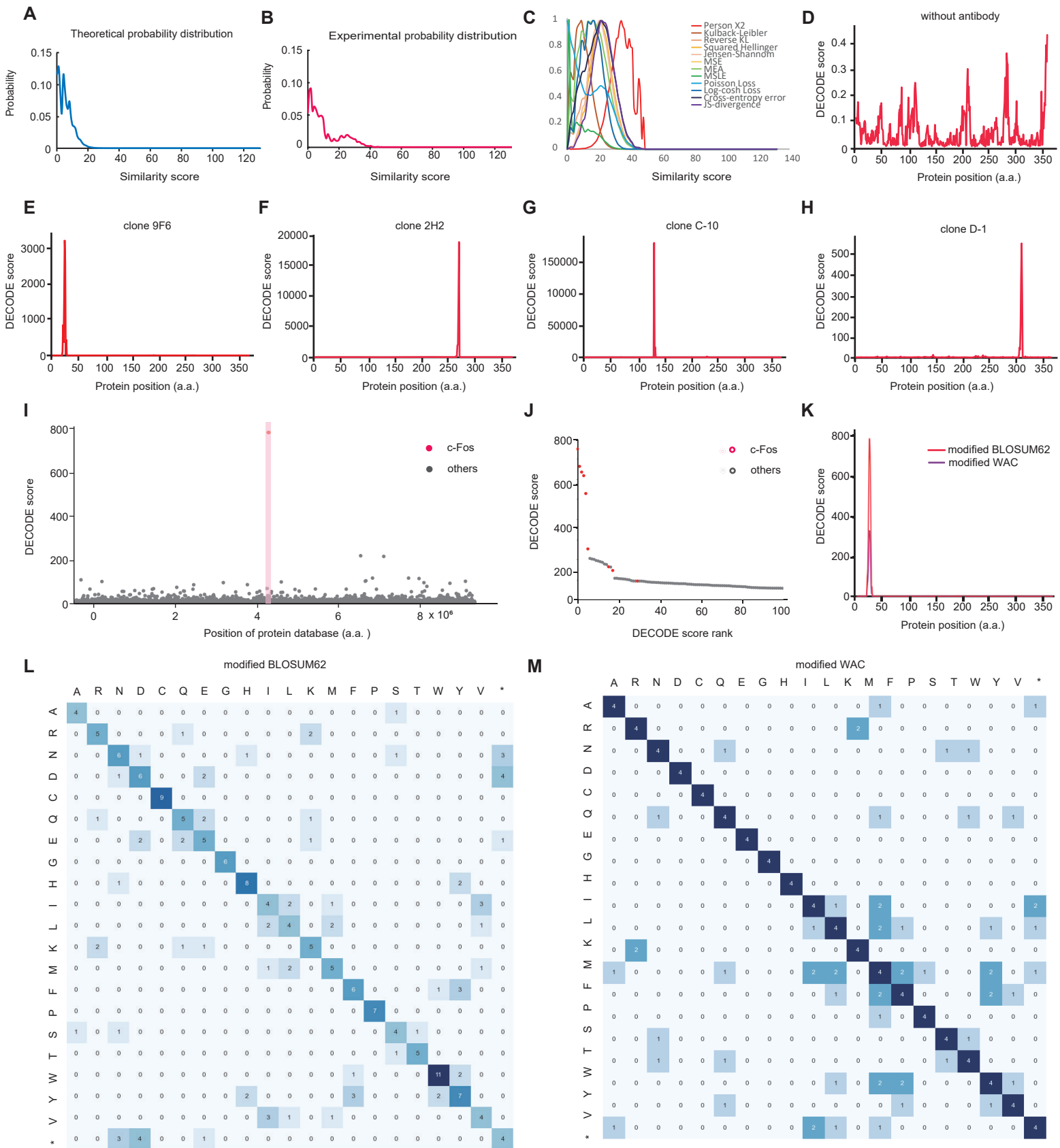

S2 Fig

Supplement: S2 Fig — (A) One of the probability distributions on each similarity score for a completely randomized DNA library. This plot shows the binding site (22–33 aa) of anti-c-fos antibody clone 9F6. (B) Distributions of the experimental probabilities of the similarity scores about clone 9F6 on the same position with A. (C) Plots of the distance at each similarity score between P and Q of the binding sites are calculated by various distance functions. (D–H) DECODE score plots on the mouse c-fos protein for monoclonal anti-c-fos antibodies and without antibodies. (I) Manhattan plot of anti-c-fos antibody clone 9F6 against all mouse proteins, which is calculated with a modified WAC table. Red dots and highlights indicate the c-fos protein. This plot was visualized with downsampled data to 1/50 using LTTB. (J) Top 100 DECODE scores of (I). Red dots indicate the c-fos protein. (K) Comparison of the DECODE score distribution on the mouse c-fos protein for anti-c-fos antibody clone 9F6 between modified BLOSUM62 and modified WAC. (L, M) Amino acid similarity tables used for DECODE analysis. Modified BLOSUM62 (L) and modified WAC (M). The data underlying for panels A–M shown in the figure can be found in S2 Data or https://doi.org/10.5281/zenodo.14286317. (PDF) [file pbio.3002707.s002.pdf]

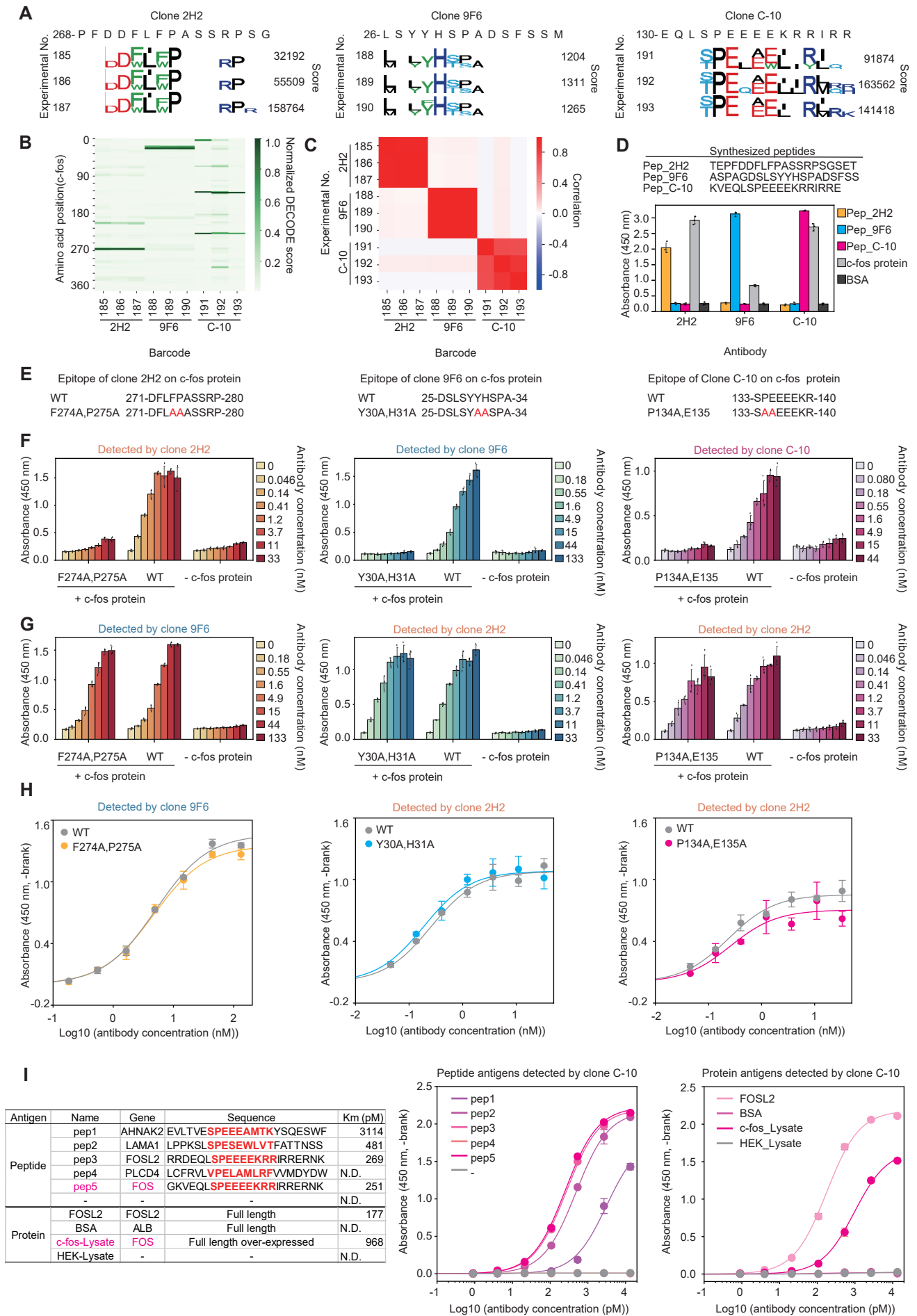

S3 Fig

Supplement: S3 Fig — (A) Epitope logos at the highest DECODE score position on the c-fos protein at the third round of DECODE selection for anit-c-fos monoclonal antibodies (9F6, 2H2, and C-10) in independent experiments (n = 3). (B) Reproducibility of the DECODE selection of 3 kinds of monoclonal anti-c-fos antibodies (clone 2H2, 9F6, and C-10) during independent experimental (n = 3). (C) Pairwise correlation of the DECODE scores between independent experiments (n = 3) or different clones. (D) The upper table shows synthesized peptides. Lower bar graph showing direct ELISA signals of A450 for each anti-c-fos antibody against upper peptides. Data are shown as actual data and means ± STD (n = 3). (E) Wild-type and double mutant c-fos protein sequences. (F, G) Direct ELISA results against wild-type and each mutant c-fos proteins by 3 anti-c-fos antibodies. Data are shown as means ± STD (n = 3). (H) Binding curves of (G). Lines represent fitting with Michaelis–Menten equation. The table shows the saturation values of each curve. (I) Direct ELISA for binding anti-c-fos antibody (C-10) to antigens derived from the 4 proteins (AHNAK2, LAMA1, FOSL2, and PLCD4) predicted to cross-react in Fig 3E. The left table lists each antigen and the Km values calculated from the binding curves. The peptide antigens were chemically synthesized based on the sequences of the regions on each protein with the highest DECODE scores. The binding curves (center) are for the peptide antigens, and the binding curves (right) are for the protein antigens. The binding curves were fitted using the Michaelis–Menten equation. Data are shown as mean ± STD (n = 3). The data underlying for panels A–I shown in the figure can be found in S2 Data or https://doi.org/10.5281/zenodo.14286317. (PDF) [file pbio.3002707.s003.pdf]

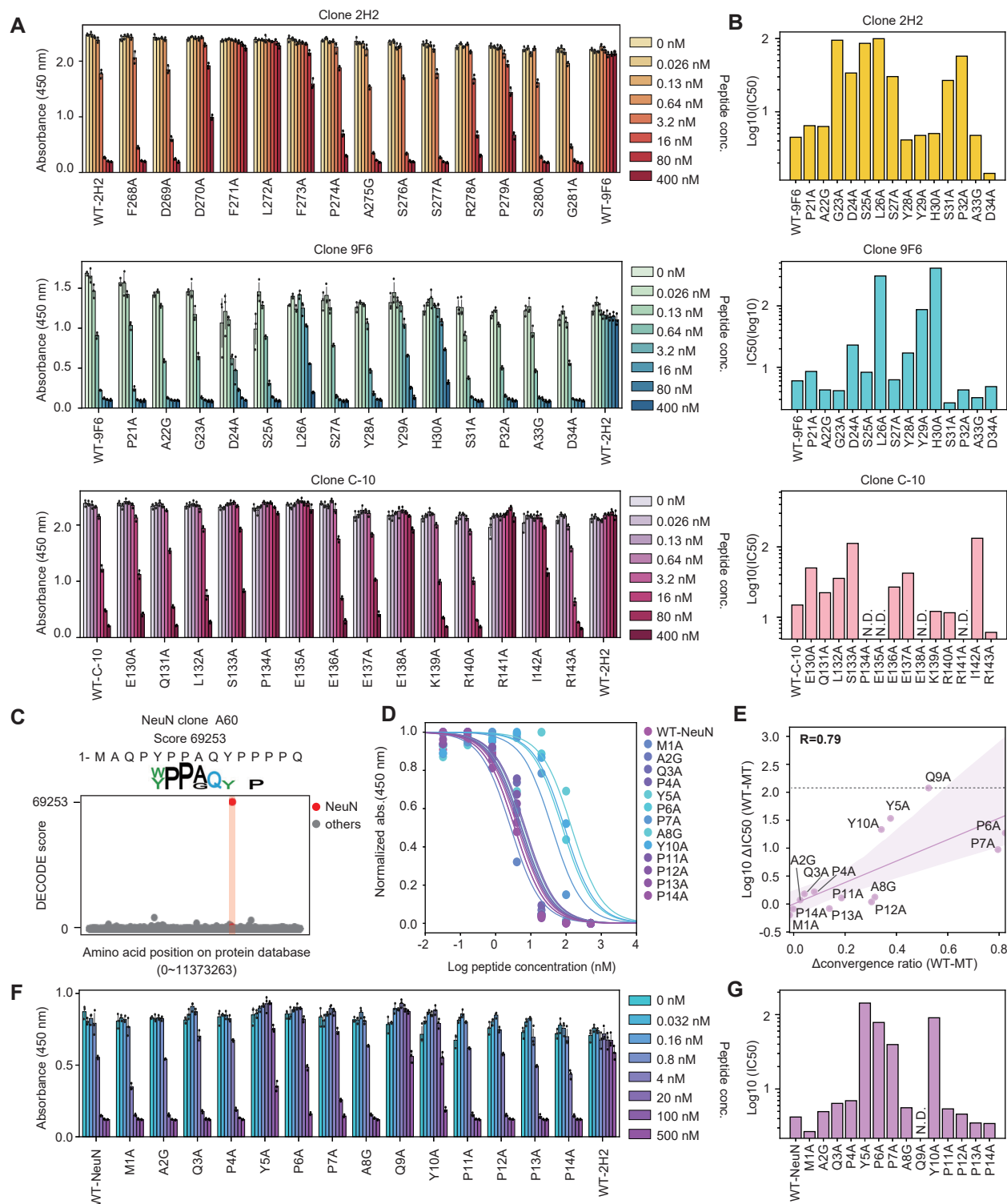

S4 Fig

Supplement: S4 Fig — (A) The bar graphs show raw data of Fig 3C. (B) The bar graph shows the logarithm of the IC50 of each mutant peptide calculated from the data in Fig 3C using the Michaelis–Menten equation. (C) Epitope sequence logo (upper) and Manhattan plot on the human protein database for anti-NeuN antibody (clone A60) (Lower). Red circles indicate NeuN protein. (D) Competitive curves of anti-NeuN antibody (clone A60) against NeuN protein inhibited by single amino acid mutant peptides at each concentration. A450s were normalized without competitive peptide conditions. Data are shown as means ± STD (n = 3). Lines represent fitting with Michaelis–Menten equation. Mutant peptide sequences are provided as S2 Table. (E) Scatter plot of the correlation about anti-NueN antibody (clone A60) between the difference in converged amino acid ratios between wild and mutant amino acids (Δconvergence ratio (WT-MT)) and the difference of IC50 calculated in S4D between mutant and wild type (Log10 ΔIC50 log (WT-MT)). Plots, lines, and shaded areas represent actual data, the regression line and the 95% confidence bounds, respectively. Spearman’s correlation coefficient was R = 0.79. (F, G) Results of the competitive ELISAs and the logarithmic IC50s of each single amino acid mutant peptide for NeuN protein for anti-NueN antibody (clone A60). Data are shown as means ± STD (n = 3). The data underlying for panels A–D and F–G shown in the figure can be found in S2 Data or https://doi.org/10.5281/zenodo.14286317. (PDF) [file pbio.3002707.s004.pdf]

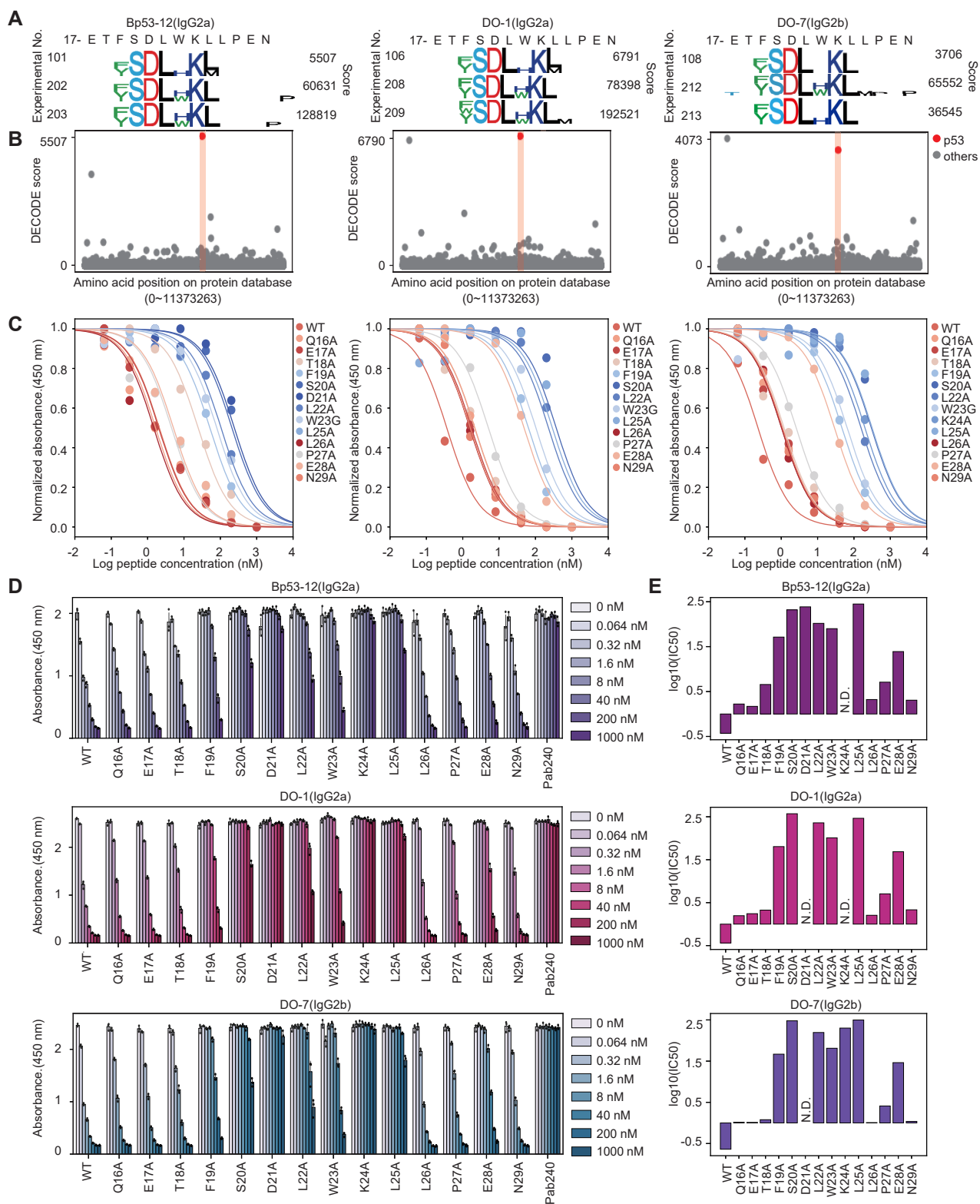

S5 Fig

Supplement: S5 Fig — (A) Sequence logos of the most converged peptide in round 3 of DECODE selection for anti-p53 monoclonal antibodies (clone Bp53-12, DO-1, and DO-7) in independent experiments (n = 3). (B) Manhattan plots of each anti-p53 antibody on the human protein database. Plots of the p53 protein are shown in red. (C) Competitive ELISAs of anti-p53 antibodies (clones Bp53-12, DO-1, and DO-7) with single amino acid mutant peptides at each concentration against p53 protein. A450 was normalized by the values without a competitive peptide. Plots indicate means ± SD of independent experiments (n = 3). Lines represent fitting to Michaelis–Menten equation. Mutant epitope peptides are provided as a peptides list (S2 Table). (D) Bar graphs show raw data of (C). (E) The bar graph shows the logarithm of the IC50 of each mutant peptide calculated from the data in (C) using the Michaelis–Menten equation. The data underlying for panels A–E shown in the figure can be found in S2 Data or https://doi.org/10.5281/zenodo.14286317. (PDF) [file pbio.3002707.s005.pdf]

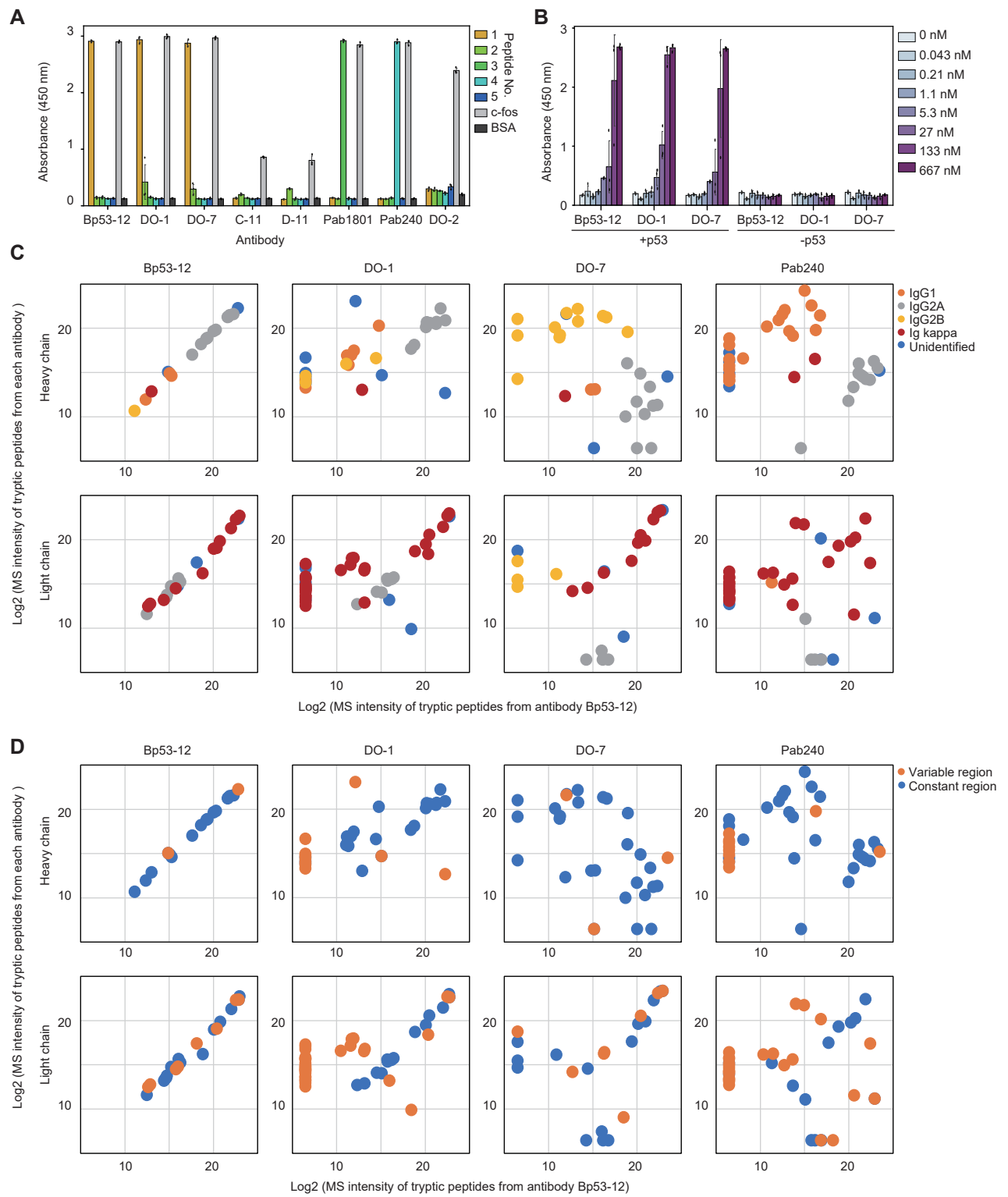

S6 Fig

Supplement: S6 Fig — (A) Direct ELISA results for anti-p53 antibodies (clones Bp53-12, DO-1, DO-7, Pab1801, Pab240, and DO-2) against each peptide shown in Fig 4C, c-fos protein and BSA. Data are shown as means ± STD (n = 3). (B) ELISA titration results in anti-p53 antibodies (clones Bp53-12, DO-1, and DO-7) against p53 protein. Data are shown as means ± STD (n = 3). (C) Scatter plots of the MS intensities of tryptic peptides derived from Bp53-12 versus 4 p53 antibodies (clones Bp53-12, DO-1, DO-7, and Pab240). Orange, gray, yellow, red, and blue circles indicate IgG, IgG2A, IgG2B, Ig kappa, and unidentified, respectively. (D) Scatter plots of the MS intensities of tryptic peptides derived from Bp53-12 versus 4 p53 antibodies (clones Bp53-12, DO-1, DO-7, and Pab240). Orange and blue circles indicate the variable region and the constant region, respectively. The data underlying for panels A–D shown in the figure can be found in S2 Data or https://doi.org/10.5281/zenodo.14286317. (PDF) [file pbio.3002707.s006.pdf]

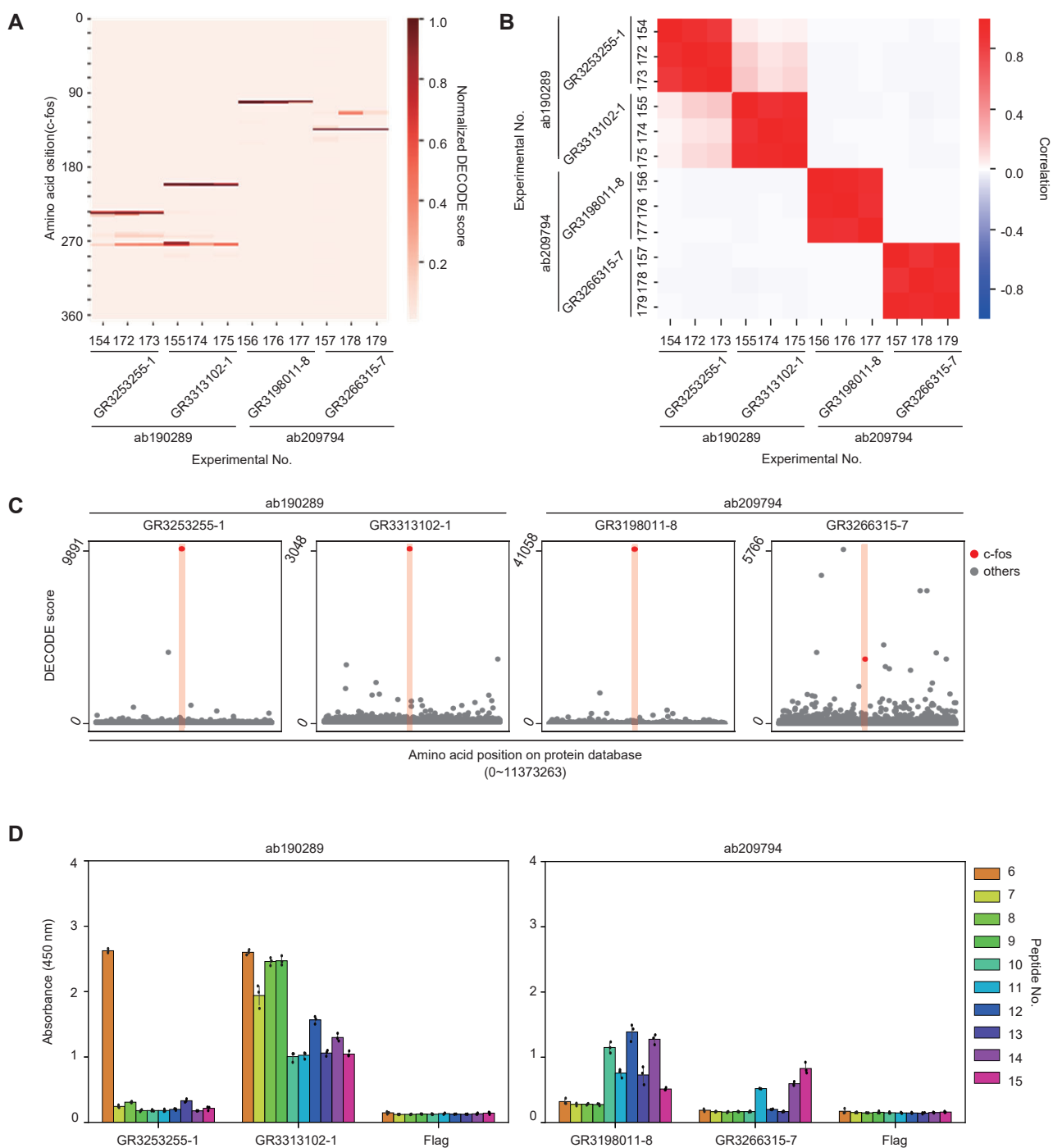

S7 Fig

Supplement: S7 Fig — (A) Verification of the anti-c-fos polyclonal antibodies at the epitope level using DECODE. Different lot products made by different individuals showed different epitope profiles. (B) Pairwise correlation of the DECODE scores between independent experiments (n = 3) and different lot products. (C) Manhattan plots of the DECODE scores on the human protein database. Each panel indicates about different lots (cat#ab190289; lot.GR323255-1, GR3313102-1, cat#ab209794; lots.GR3198011-8, GR3266315-7) at third DECODE selection round. Red circles indicate c-fos protein. (D) ELISA results of anti-c-fos polyclonal antibodies against each epitope peptide. Data are shown as means ± STD (n = 3). The data underlying for panels A–D shown in the figure can be found in S2 Data or https://doi.org/10.5281/zenodo.14286317. (PDF) [file pbio.3002707.s007.pdf]

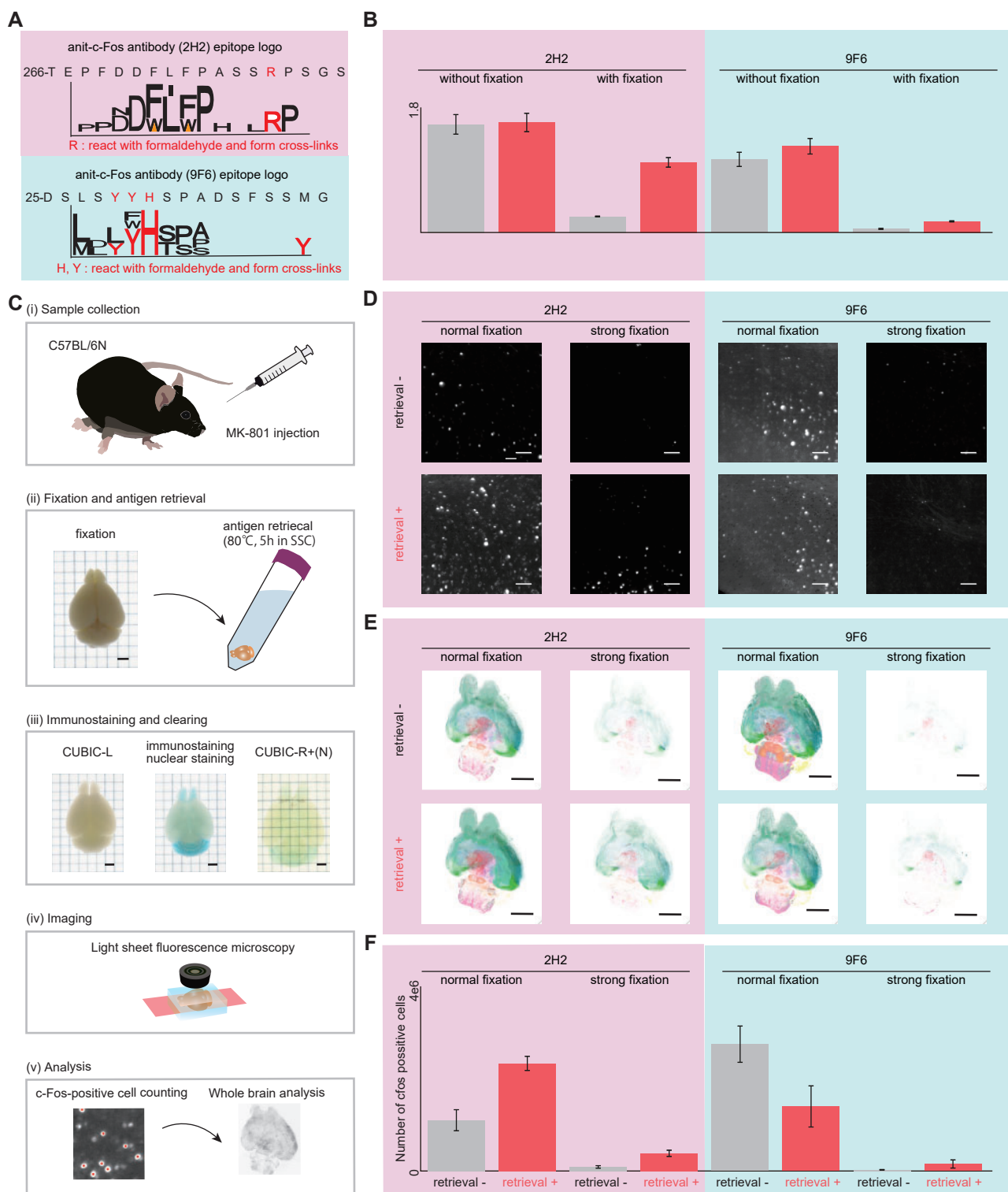

S8 Fig

Supplement: S8 Fig — (A) Sequence logos of the most converged peptide in round 3 of the DECODE analysis for anti-c-fos monoclonal antibodies (clone 2H2 and 9F6). Amino acids indicated red form irreversible methylene bridge with amine group by formalin or PFA fixation. (B) Verification by ELISA of antigenicity changes of each anti-c-fos antibody depending on the fixation strength and the presence or absence of antigen retrieval. Data are shown as means ± STD (n = 6). (C) Workflow of whole-brain immunostaining using anti-c-fos monoclonal antibodies (clone 2H2 or 9F6) and whole-brain analysis of c-fos positive cells. (i) Injection of the drug (MK-801), perfusion fixation, and dissection of the brain. (ii) Antigen retrieval within saline sodium citrate buffer (SSC) at 80°C for 5 h. Strongly fixed samples were mimicked by PFA + 100 mM Gly. (iii) Tissue clearing and staining with CUBIC-HV method. (iv) 3D imaging with a high-resolution light-sheet fluorescence microscope. (v) Cell detection counting of c-fos positive cells across the whole brain. (D) Representative single-plane brain images of the c-fos signal without (upper) or with (lower) antigen retrieval. Scale bars, 100 μm. (E) Magnified images of the hippocampus. Scale bars, 2 mm. (F) Cell number of c-fos positive cells. Data are shown as means ± STD (n = 3). The data underlying for panels A, B, and F shown in the figure can be found in S2 Data or https://doi.org/10.5281/zenodo.14286317. (PDF) [file pbio.3002707.s008.pdf]

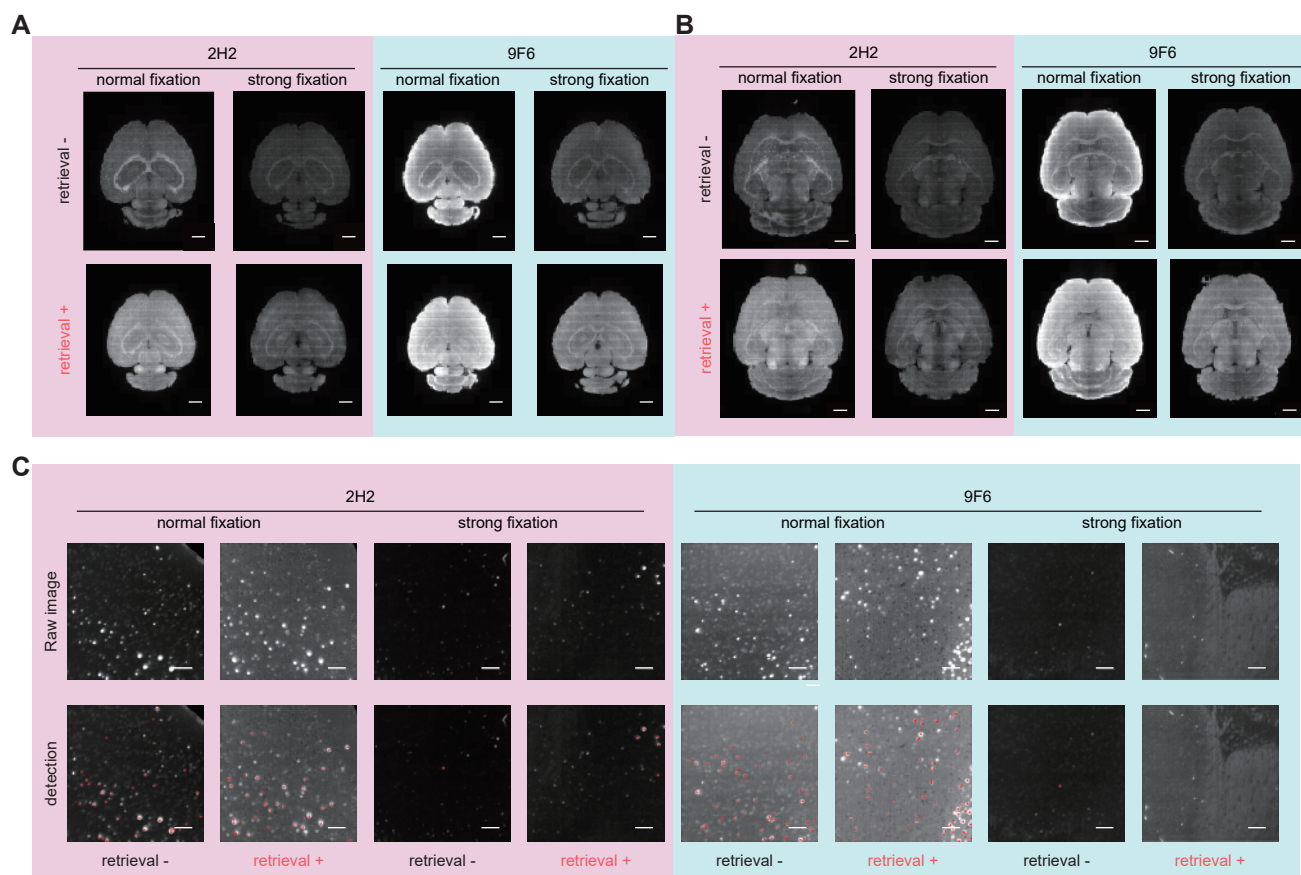

S9 Fig

Supplement: S9 Fig — (A) Magnified images of the cortex. Scale bars, 2 mm. (B) Detection of the c-fos positive cells. The red dot indicated detected cells. Scale bars, 2 mm. (C) Other depth images of a single plate in S8 Fig. Scale bars, 100 μm. (PDF) [file pbio.3002707.s009.pdf]

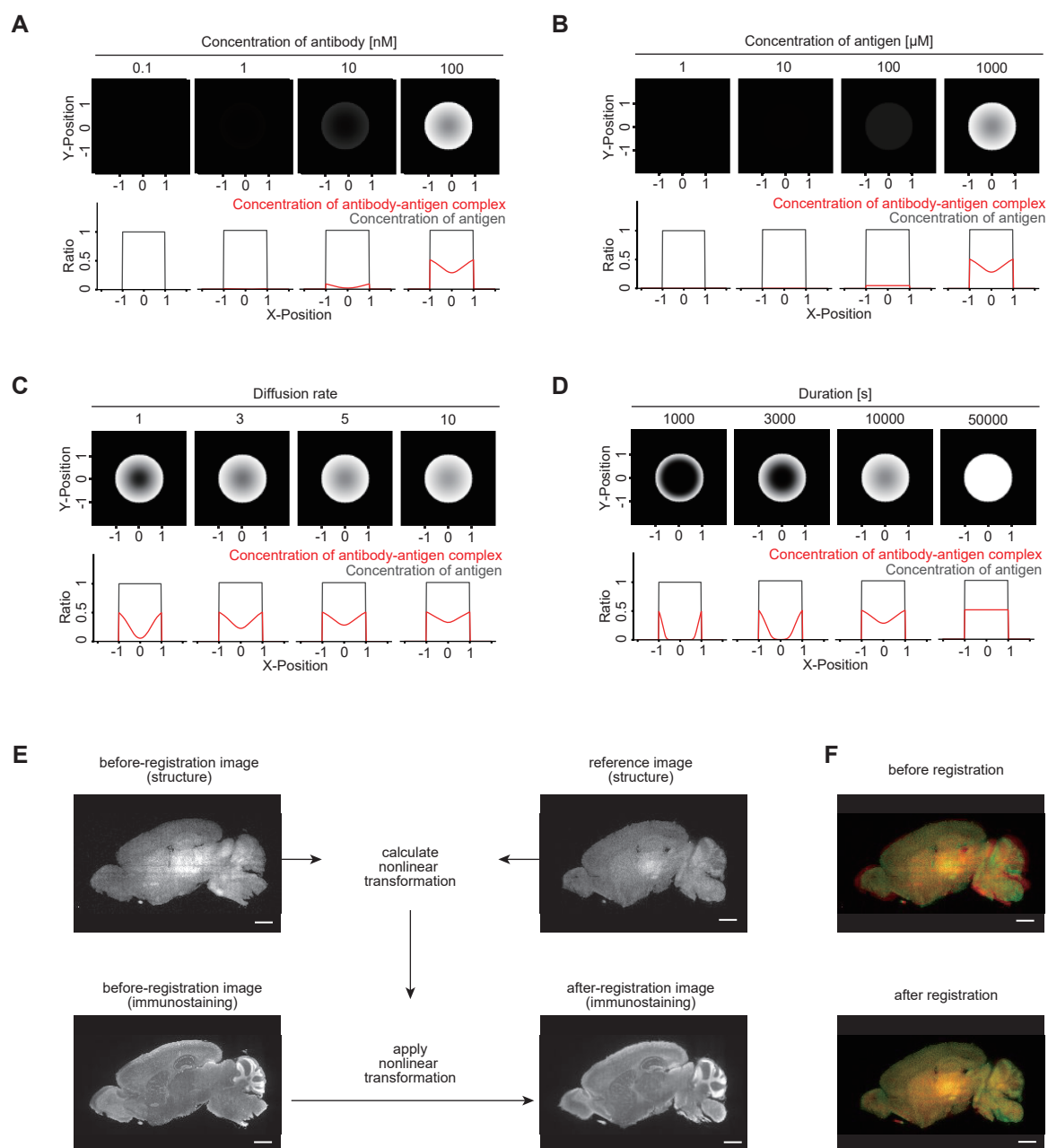

S10 Fig

Supplement: S10 Fig — (A–D) Simulation of antibody penetration speed changes due to changes in each parameter. The upper panels show staining patterns. The lower line graph shows the concentration of antibody-antigen complex (red) and total antigen (gray) in the cross-sectional region at Y = 0. (E) Schematic of the transformation of whole-brain images for comparative analysis. The nonlinear transformation is calculated using the before-registration structure image (upper left) and the reference structure image using auto fluorescence at the GFP channel (upper right). The nonlinear transformation is applied to the before-registration immunostaining image (lower left) to output the after-registration immunostaining image (lower right). Scale bars, 2 mm. (F) The upper panel shows the merged image before registration (red, before-registration structure image; green, reference structure image). The lower panel shows the merged image after registration (red, after-registration structure image; green, reference structure image). Scale bars, 2 mm. The data underlying for panels A–D shown in the figure can be found in S2 Data or https://doi.org/10.5281/zenodo.14286317. (PDF) [file pbio.3002707.s010.pdf]

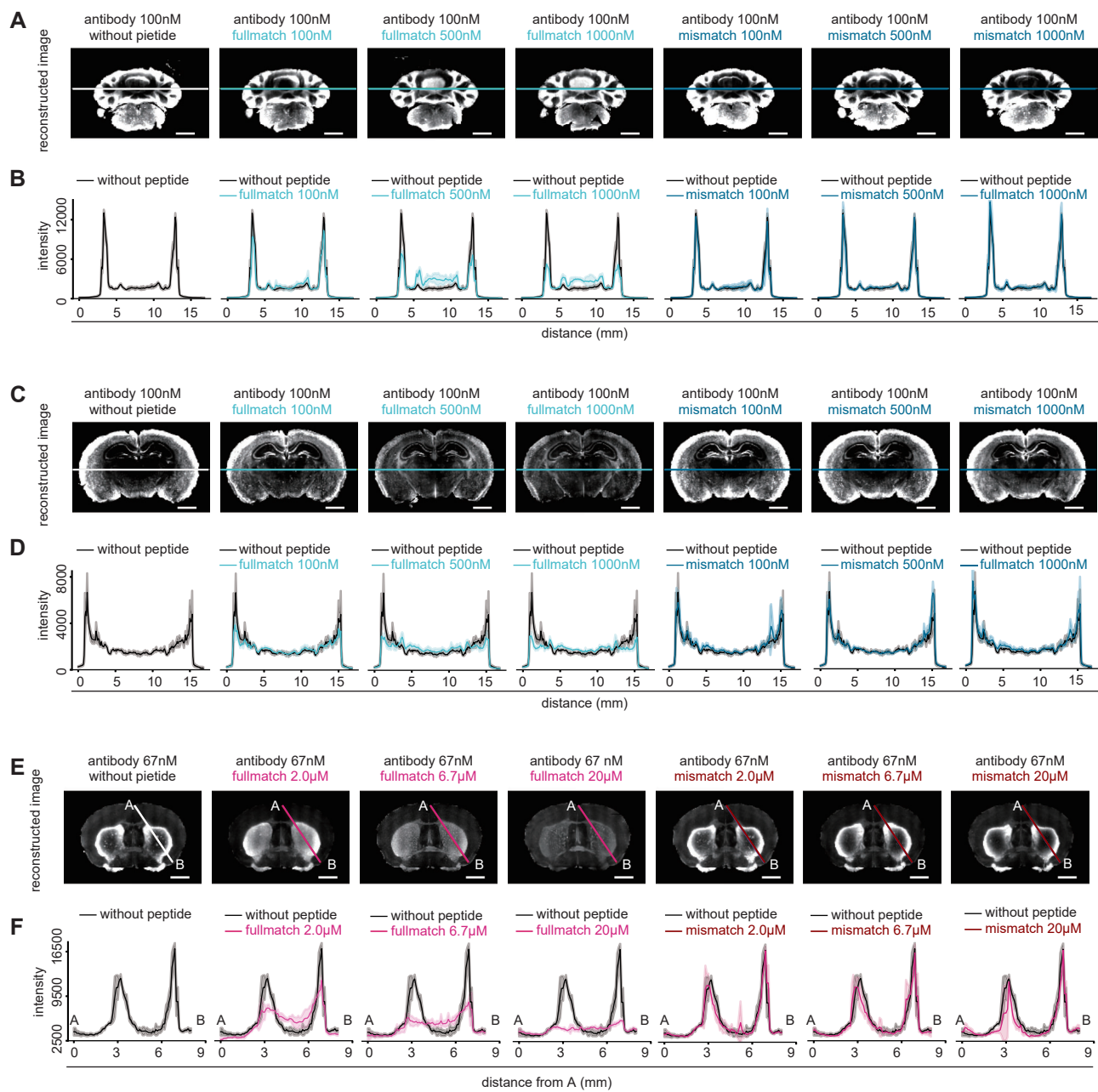

S11 Fig

Supplement: S11 Fig — (A) Immunostained images stained by anti-NeuN antibody (clone A60) with or without epitope peptide of the coronal plane of the registered brain. The color lines in the images show cross-sectional locations. Scale bars, 2 mm. (B) Mean intensities of the cross-section by the line in (A). (C, D) Immunostained images of the other position of S10A Fig. (E) Immunostained images stained by anti-TH antibody (clone EP1532Y) with or without epitope peptide of the coronal plane of the registered brain. The color lines in the images show cross-sectional locations. Scale bars, 2 mm. (F) Mean intensities of the cross-section by the line in S10E Fig. Data are shown as means ± STD (n = 3). The data underlying for panels B, D, and F shown in the figure can be found in S2 Data or https://doi.org/10.5281/zenodo.14286317. (PDF) [file pbio.3002707.s011.pdf]

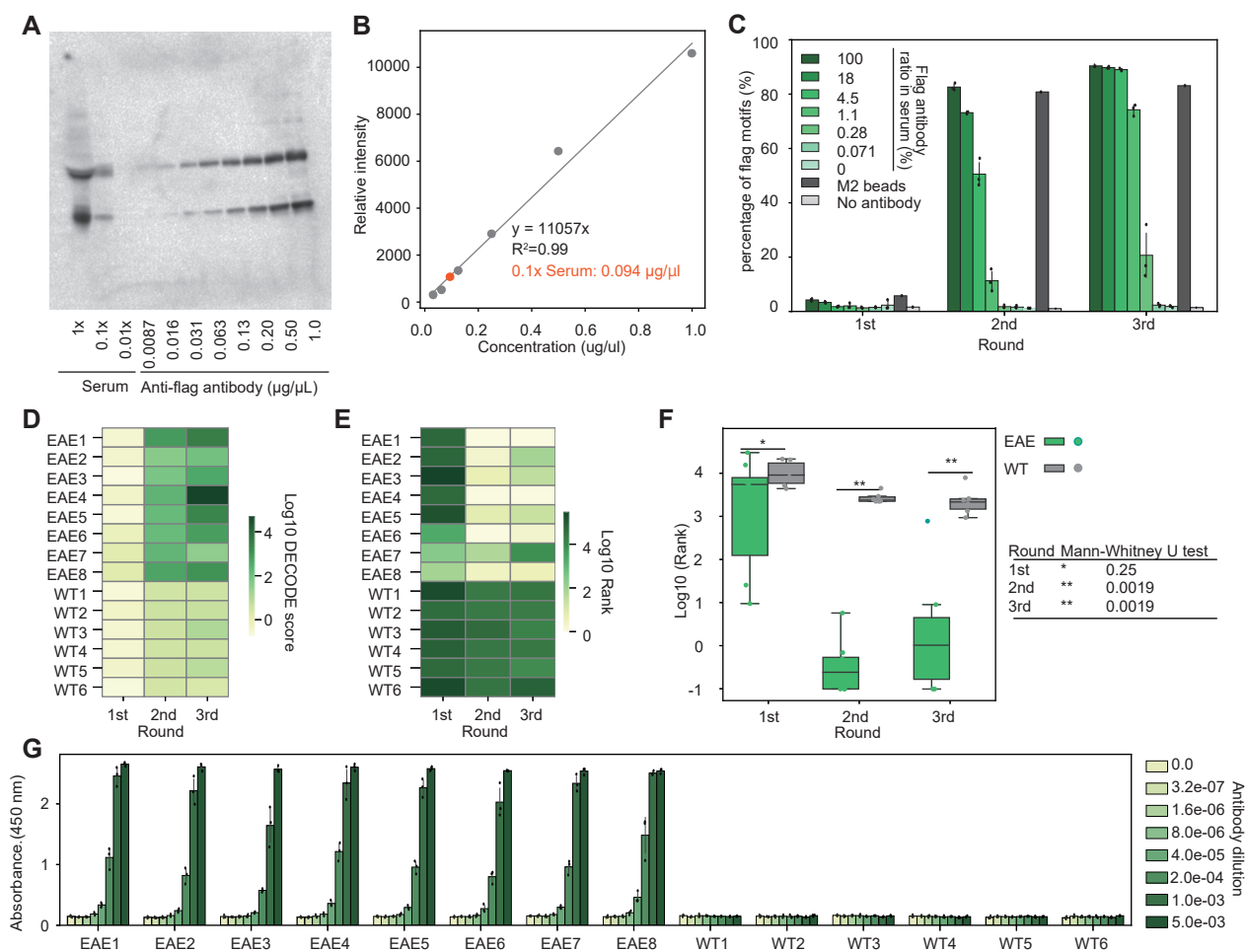

S12 Fig

Supplement: S12 Fig — (A) Western blot of mouse serum antibodies and anti-Flag M2 antibody detected by anti-mouse IgG-HRP antibody. (B) The standard curve shows the relative intensity of mouse IgG derived from dilution series of anti-Flag M2 antibodies as shown in (A). The equation was obtained by linear approximation (Intercept = 0, R2 = 0.99). The IgG concentration in 1/10 diluted wild-type mouse serum was determined to be 0.094 μg/μl. (C) Bar plots of Fig 6B. (D) The highest DECODE score (log10) in the MOG protein sequence in each DECODE selection round. (E) The highest rank of the epitope on the MOG protein in the mouse protein database in each DECODE selection round. (F) Box-whisker plot showing the maximum rank on the MOG protein in the mouse protein database comparing EAE (green, n = 8) and healthy (gray, n = 6). P values were obtained by Mann–Whitney’s U test (* = 0.25, ** = 0.0019). (G) Raw data of Fig 6G is shown. The data underlying for panels B–G shown in the figure can be found in S2 Data or https://doi.org/10.5281/zenodo.14286317. (PDF) [file pbio.3002707.s012.pdf]

**A**

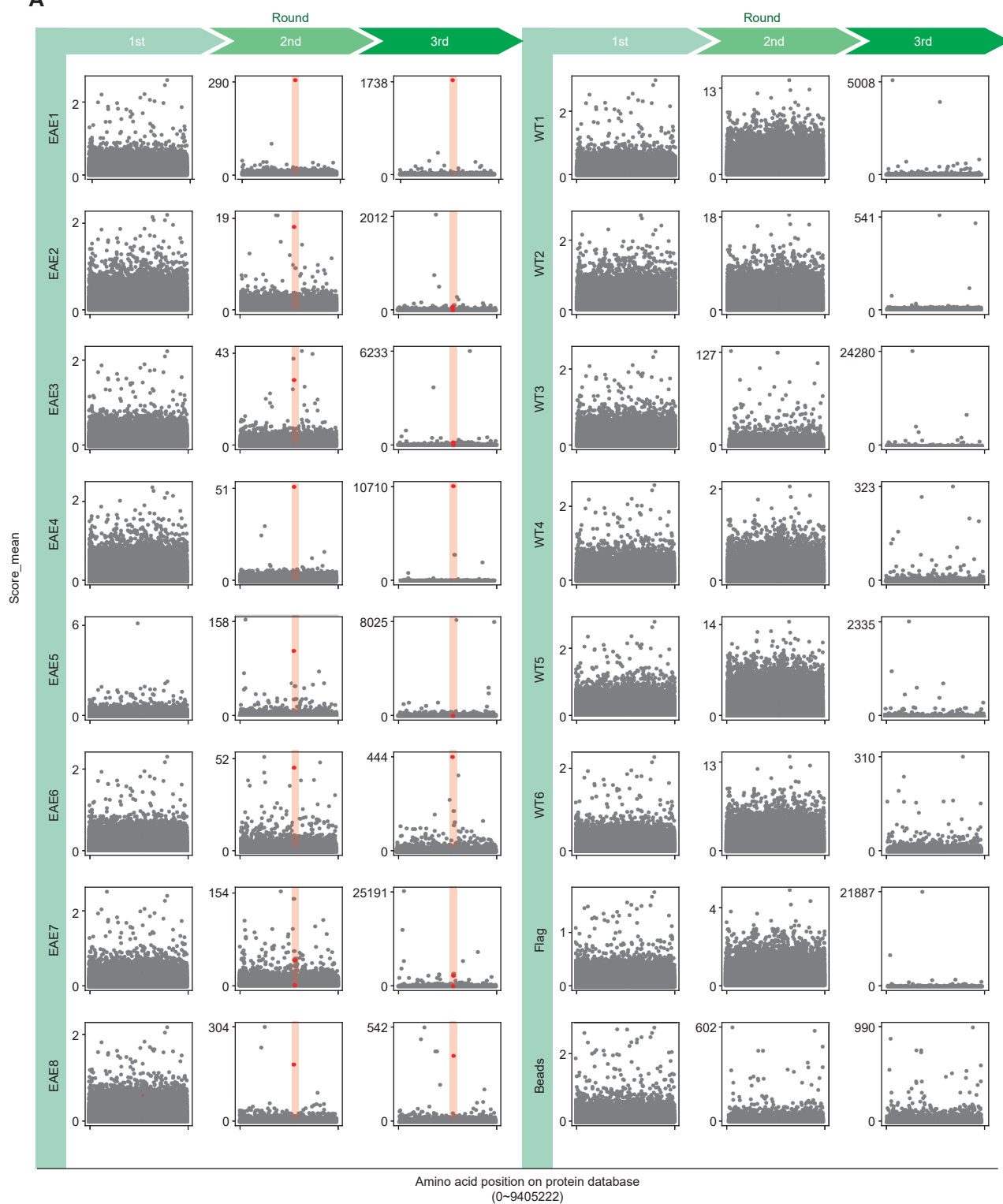

S13 Fig

Supplement: S13 Fig — (A) Manhattan plot of the DECODE scores on the mouse protein database. Red circles indicate MOG protein. The data underlying for panels A shown in the figure can be found in S2 Data or https://doi.org/10.5281/zenodo.14286317. (PDF) [file pbio.3002707.s013.pdf]

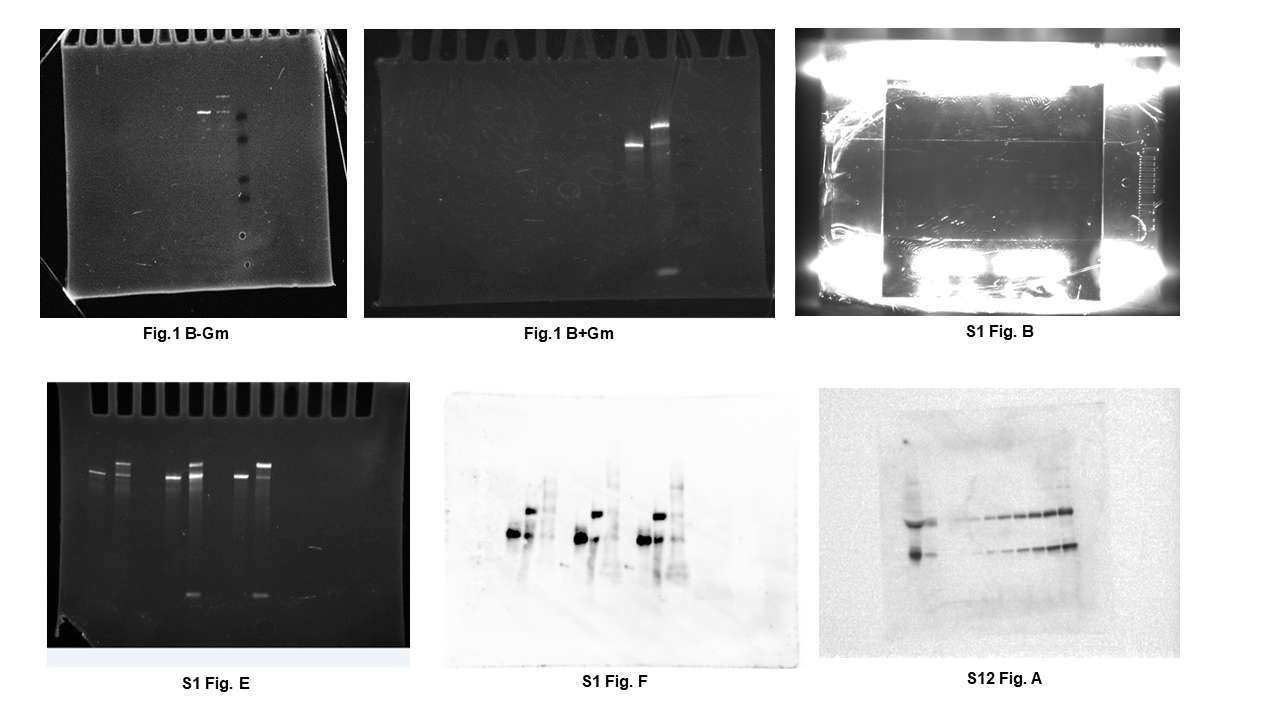

Supplement: S1 Raw Images — (TIF) [file pbio.3002707.s020.tif]
