# Supplementary material for: DECODE enables high-throughput mapping of antibody epitopes at single amino acid resolution
Source: PLoS Biol. 2025 Jan 23;23(1):e3002707. doi: 10.1371/journal.pbio.3002707 (PMC11756784; doi:10.1371/journal.pbio.3002707)
Supplement: S1 Table — (PDF) [file pbio.3002707.s014.pdf]

S1 Table. Results of comprehensive epitope analysis by DECODE

experimental no.	NGS barcode	target	clone	clonality	maker	cat#	lot#	species reactivity*	RRID	DECODE	qPCR (Ct value)** 1st	2nd	3rd
1	97	AFP	39	mono	Santa Cruz	sc-130302	K0618	human,mouse,rat	AB_2223934		22	20	14
2	67	ALDH1L1	YY8	mono	Santa Cruz	sc-100497	G2618	human,mouse,rat	AB_2224180		20	20	15
3	79	ASH2L	EPR13107(B)	mono	Abcam	ab176334	GR137013-2	human,mouse,rat			21	22	17
4	20	Beta3-tubulin	TU20	mono	Novusbio	NB600-1018	53113	human,mouse,rat,cat	AB_10001615		20	13	6
5	78	Beta3-tubulin	D71G9	mono	CST	5568S	6	human,mouse,rat	AB_10694505		23	21	8
6	121	BMAL1	B-1	mono	Santa Cruz	sc-365645	J1018	human	AB_10841724		22	19	10
7	133	BMAL2	C-7	mono	Santa Cruz	sc-376287	E2118	human,mouse,rat	AB_10988202		21	22	16
8	110	C1q	1A4	mono	Santa Cruz	sc-53544	G0617	human	AB_1119798		19	12	7
9	1	Calbindin D28K		poly	Sigma	C2724	093M4801	rat	AB_258818		21	13	9
10	15	Camk2b		poly	Sigma	HPA026307	R27007	human,mouse	AB_1845941		19	21	10
11	157	caspase-3	31A1067	mono	Santa Cruz	sc-56053	C1419	human	AB_781826		19	21	11
12	169	caspase-3	E-8	mono	Santa Cruz	sc-7272	A1519	human	AB_626803		21	22	16
13	181	caspase-3 p11	C-6	mono	Santa Cruz	sc-271759	L1917	human	AB_10709891		18	21	10
14	98	caspase-3 p17	B-4	mono	Santa Cruz	sc-271028	L1718	human	AB_10609624		19	15	5
15	6	c-fos		poly	Millipore	ABE457		human, rat	AB_2631318		22	20	10
16	21	c-fos		poly	sigma	HPA018531	E106326	human, rat	AB_1846576		20	21	16
17	45	c-fos	2H2	mono	abcam	ab208942	GR3264447-5	human,mouse,rat	AB_2747772		20	12	8
18	65	c-fos		poly	Sigma	HPA018531	E105612	human	AB_1846576		22	19	11
19	69	c-fos	9F6	mono	CST	2250S	9	human,mouse,rat	AB_2247211		20	20	10
20	77	c-fos	2H2	mono	Abcam	ab208942	GR310711-1	human,mouse,rat	AB_2747772		21	15	8
21	89	c-fos		poly	Santa-cruz	sc-52G	K1314	human	AB_2629503		19	20	8
22	122	c-fos	C-10	mono	Santa Cruz	sc-271243	F2119	human	AB_10610067		21	18	6
23	134	c-fos	D-1	mono	Santa Cruz	sc-8047	C2019	human	AB_627253		19	15	6
24	146	c-fos	E-8	mono	Santa Cruz	sc-166940	A2319	human	AB_10609634		22	20	10
25	62	ChAT		poly	Millipore	AB144	2603400	human,mouse,rat	AB_90650		22	18	11
26	74	ChAT	EPR16590	mono	abcam	ab178850		mouse,rat	AB_2721842		23	18	12
27	158	choactase	E-7	mono	Santa Cruz	sc-55557	K2018	human,mouse,rat	AB_2291743		22	19	16
28	170	Clock	C-8	mono	Santa Cruz	sc-271603	B2118	human,mouse,rat	AB_10659237		20	14	5
29	2	Copeptin		poly	Santa Cruz	sc-7812	J0604	mouse	AB_2061966		22	15	12
30	55	CREB1	48H2	mono	CST	9197	16	human,mouse,rat	AB_331277		19	17	23
31	99	CRY1	W-L5	mono	Santa Cruz	sc-101006	L2818	human	AB_2260923		19	20	9
32	182	CRY1	H-12	mono	Santa Cruz	sc-393466	L1818	human	AB_2917985		19	15	7
33	66	CSPG	Cat-315	mono	Millipore	MAB1581	3004121	rat	AB_94270		20	19	9
34	9	DAT		poly	sigma	HPA013602	A97032	human	AB_1847484		23	15	7
35	50	Dat	6V-23-23	mono	abcam	ab128848		mouse,rat	AB_2665470		20	15	7
36	111	DAT	6-5G10	mono	Santa Cruz	sc-32258	B1618	human	AB_627400		18	22	16
37	123	DAT	6-8D6	mono	Santa Cruz	sc-32259	A2919	human	AB_627402		22	24	12
38	3	Dbh	EPR20385	mono	abcam	ab209487		mouse,rat,human	AB_2892178		21	19	8
39	135	DBH	A-9	mono	Santa Cruz	sc-365710	E2417	human	AB_10844004		19	17	6
40	147	DBH	DBH 41	mono	Santa Cruz	sc-47707	K0917	human,mouse,rat	AB_627407		20	19	8
41	159	DDC	8.00E+08	mono	Santa Cruz	sc-293287	A2218	human,mouse,rat			22	20	18
42	171	E4BP4	A-9	mono	Santa Cruz	sc-74415	D2717	human,mouse,rat	AB_1123159		19	18	6
43	183	E4BP4	C-6	mono	Santa Cruz	sc-374451	B1618	human,mouse,rat	AB_10989421		19	19	17
44	100	Ep-CAM	9C4	mono	Santa Cruz	sc-21792	B1419	human	AB_627529		22	19	11
45	112	Ep-CAM	C-10	mono	Santa Cruz	sc-25308	B0619	human	AB_627531		21	19	8
46	124	Ep-CAM	EBA-1	mono	Santa Cruz	sc-66020	C2818	human,mouse,rat	AB_2098654		20	21	14

species reactivity*: This refers to information provided by the antibody manufacturers on their websites.
qPCR (Ct value)**: The cDNA-peptide quantification results for the positive control sequence (mc1') that specifically binds to the anti-FLAG antibody showed a Ct value of 7-9.

47	136	Ep-CAM	G8.8	mono	Santa Cruz	sc-53532	H1617	mouse	AB_2277892		19	22	17
48	148	Fibroblast Marker	ER-TR7	mono	Santa Cruz	sc-73355	L1218	human,mouse	AB_1122890		18	22	9
49	160	GAD-65	A-3	mono	Santa Cruz	sc-377145	A1618	human	AB_2619725		19	21	11
50	172	GAD-65/67	C-9	mono	Santa Cruz	sc-365180	D0418	human	AB_10710523		22	23	15
51	25	GAD67	1G10.2	mono	Millipore	MAB5406		human,mouse,rat	AB_2278725		22	20	16
52	184	GAD-67	F-6	mono	Santa Cruz	sc-28376	L0718	human	AB_627650	N.D.	20	22	17
53	101	Gastric Lipase	H-1	mono	Santa Cruz	sc-390750	I0617	mouse			20	20	10
54	113	GATA-3	HG3-31	mono	Santa Cruz	sc-268	H0718	human	AB_2108591		22	21	13
55	75	GFAP	MO389	mono	MBL	D097-3	12	human	AB_591769		20	22	15
56	87	GFAP		poly	Sigma	G9269		human,rat	AB_477035		20	18	11
57	125	GFAP	2A5	mono	Santa Cruz	sc-65343	I2617	human,mouse,rat	AB_783553		18	23	15
58	137	GFAP	2.00E+01	mono	Santa Cruz	sc-33673	C1219	human,mouse,rat	AB_627673		20	21	8
59	149	GFAP	F-2	mono	Santa Cruz	sc-166481	B0518	human,mouse,rat	AB_2294569		21	18	6
60	161	GFAP	F-7	mono	Santa Cruz	sc-166458	A1618	human	AB_2109811		21	14	6
61	63	GFAP-Cy3	G-A-5	mono	Sigma	C9205	conj	human,rat	AB_476889		22	20	9
62	37	Glutamic Acid Decarboxylase	9A6	mono	MBL	M018-3	24	mouse,rat	AB_591728		21	16	11
63	49	Glutaminase	EP7212	mono	Abcam	ab156876		human	AB_2721038		21	18	10
64	185	Golgi Marker	AE-6	mono	Santa Cruz	sc-58770	L2018	human	AB_2113343		21	22	10
65	44	HES1	NM1	mono	MBL	D134-3	16		AB_590682		16	20	15
66	19	Histone H2A	L88A6	mono	CST	3636S	1	human,mouse,rat,mk	AB_2118801		15	15	6
67	7	Histone H2B	D2H6	mono	CST	12364S	1	human,mouse,rat	AB_2714167		22	19	17
68	31	Histone H3	D2B12	mono	CST	4620	12	human,mouse	AB_1904005		21	14	6
69	90	Histone H3	D1H2	mono	CST	4499S	9	human,mouse,rat,mk	AB_10544537		19	18	7
70	8	Iba1	A635	mono	Wako	013-26471	PTM6693	mouse,rat	AB_2687911		18	16	7
71	28	Iba1		poly	Wako	019-19741		human,mouse,rat	AB_839504		20	18	22
72	91	Iba1	NCNP24	mono	Wako	016-26721	PTK2881	mouse,rat,marmo	AB_2811160		19	20	11
73	102	Iba1	1022-5	mono	Santa Cruz	sc-32725	B0819	human,mouse,rat	AB_667733		19	22	16
74	138	IgE	154/102	mono	Santa Cruz	sc-53346	C1717	human	AB_783970		19	20	17
75	150	IgG	3.00E+08	mono	Santa Cruz	sc-69786	B2019		AB_1124809		18	17	5
76	162	IgG	D-1	mono	Santa Cruz	sc-515946	J2317	human			20	23	18
77	174	IgM	R1/69	mono	Santa Cruz	sc-53347	G1217	human	AB_672096		19	22	14
78	186	Ki-67	Ki-67	mono	Santa Cruz	sc-23900	A2319	human	AB_627859		20	14	7
79	18	LRPAP-1		poly	Sigma	HPA008001	R02480	human,mouse,rat	AB_1853292		21	15	8
80	52	MAP2		poly	Sigma	HPA012828	A96874	human	AB_1853946		22	19	16
81	127	MHC class I	ER-HR52	mono	Santa Cruz	sc-59199	C2718	mouse	AB_1126186		21	20	13
82	139	MHC class I	F-3	mono	Santa Cruz	sc-55582	B1618	human	AB_831547		20	22	16
83	151	MHC class I	W6/32	mono	Santa Cruz	sc-32235	J23517	human	AB_627934		20	13	5
84	175	MHC class II	Y-Ae	mono	Santa Cruz	sc-32247	K2817	mouse	AB_627939		19	22	17
85	104	MOG	D-2	mono	Santa Cruz	sc-376138	J2717	human,mouse,rat	AB_10989782		21	21	15
86	187	MOG	D-10	mono	Santa Cruz	sc-166172	J2017	human,mouse,rat	AB_2145540		20	16	6
87	68	MPZ		poly	CST	PD046	1				15	19	9
88	116	Mucin 16	C-6	mono	Santa Cruz	sc-365002	A2617	human	AB_10708400		22	21	16
89	128	Myeloid lineage	OX82	mono	Santa Cruz	sc-53093	J2116	mosue,rat	AB_630017		18	18	9
90	57	NeuN	ERP12763	mono	abcam	ab177487	GR249899-58	human,mouse,rat	AB_2532109		22	20	18
91	61	NeuN	A60	mono	Millipore	MAB377	2763860	human,mouse,rat	AB_2298772		22	12	6
92	73	NeuN		poly	Millipore	ABN78	2885346	human,mouse,rat	AB_10807945		20	21	19
93	92	NeuN	A60	mono	Merck Millipore	MAB377	2829834	human,mouse,rat	AB_2298772		21	14	8
94	64	Neurofilament(PAN)	FNP7	mono	invitrogen	180171Z	1634056A	human,rat	AB_86694		20	19	21
95	140	NF-H	RNF402	mono	Santa Cruz	sc-32729	B0218	human,mouse,rat	AB_628001		22	16	7
96	4	Olig2		poly	IBL	18953	1D-104	human	AB_494617		18	18	6

97	16	Olig2	EPR2673	mono	Abcam	ab109186	GR210294-11	human,mouse,rat	AB_10861310		23	21	17
98	152	OLIG2	1G11	mono	Santa Cruz	sc-293163	C0519	human,mouse,rat			21	20	15
99	164	OLIG2	H-10	mono	Santa Cruz	sc-515947	K0218	human,mouse,rat			21	18	9
100	176	Orexin-A	KK09	mono	Santa Cruz	sc-80263	L1517	human,mouse,rat	AB_1126868		20	20	8
101	105	p53	Bp53-12	mono	Santa Cruz	sc-263	E2118	human,mouse,rat	AB_628084		20	17	6
102	106	p53	Pab 240	mono	Santa Cruz	sc-99	K2817	human,mouse,rat	AB_628086		20	21	14
103	117	p53	C-11	mono	Santa Cruz	sc-55476	B0119	human,mouse,rat	AB_630061		20	21	15
104	118	p53	Pab 246	mono	Santa Cruz	sc-100	J0617	mouse,rat	AB_628087		22	15	6
105	129	p53	D-11	mono	Santa Cruz	sc-17846	L1417	human,mouse,rat	AB_628081		21	22	15
106	141	p53	DO-1	mono	Santa Cruz	sc-126	C1419	human,mouse,rat	AB_628082		19	20	16
107	153	p53	DO-2	mono	Santa Cruz	sc-53394	J0617	human,mouse,rat	AB_785038		20	14	5
108	165	p53	DO-7	mono	Santa Cruz	sc-47698	B2019	human	AB_628083		19	20	18
109	177	p53	pAb 122	mono	Santa Cruz	sc-56182	A2218	human,mouse,rat	AB_785041		20	13	5
110	188	p53	A-1	mono	Santa Cruz	sc-393031	A3119	human,mouse,rat			21	16	7
111	189	p53	Pab 1801	mono	Santa Cruz	sc-98	K1218	human,mouse,rat	AB_628085		21	20	16
112	39	p75NTR	25-8	mono	MBL	D297-3	1	mouse	AB_10794310		21	22	10
113	130	pan CEA	H-8	mono	Santa Cruz	sc-48364	C1218	human	AB_627155		18	21	12
114	13	Parvalbumin(PV)	PV235	mono	Swant	235	10-11 (F)	human,mouse	AB_10000343		21	19	20
115	142	Per1	E-8	mono	Santa Cruz	sc-398890	I1918	human,mouse,rat			20	20	8
116	154	Per2	C-6	mono	Santa Cruz	sc-377290	A0319	human			20	18	7
117	40	phospho-neurofilament	SMI31 P	mono	Biolegend	801601	B200659	human,mouse,rat	AB_2564641		20	20	12
118	30	PKCa/PRKCA1		poly	LSBio	LS-C164546	75278	human,mouse			19	7	5
119	42	PKCa/PRKCA1	MC5	mono	Novus	NB600-201	A-2	human,mouse,rat	AB_10003372		22	19	14
120	166	PSA	A67-B/E3	mono	Santa Cruz	sc-7316	H2718	human	AB_2279058		21	22	16
121	41	PSD95	6G6-1C9	mono	Abcam	ab2723	GR299294-4	mouse,rat	AB_303248		20	19	15
122	53	PSD95	D27E11	mono	CST	3450S	2	human,mouse,rat	AB_2292883		21	17	9
123	27	RAP1GAP		poly	Sigma	HPA001922	R03996	human,mouse	AB_1079743		20	14	9
124	32	Reelin(CR-50)	RE-3B9(R3B9)	mono	MBL	D223-3	15	mouse	AB_843523		19	19	9
125	178	Rev-erbα	E-12	mono	Santa Cruz	sc-393215	C2018	human			21	22	15
126	190	Rev-erbα	RS-14	mono	Santa Cruz	sc-100910	A0319	human	AB_2154647		21	22	12
127	119	RORγ	D-4	mono	Santa Cruz	sc-365476	I1817	human	AB_10847088		20	18	8
128	54	Semaphorin 3A	A-12	mono	Santa Cruz	sc-74554	F0817	human,mouse,rat	AB_2254335		21	16	9
129	29	Synapsin-1		poly	Millipore	AB1543P	2195992	human,mouse,rat	AB_90757		20	20	11
130	76	Synaptophysin	171B5	mono	MBL	D073-3	13	human,mouse,rat	AB_592778		20	12	6
131	5	Synaptotagmin1 (Rat)	3F10	mono	MBL	D157-3		rat	AB_592779		21	17	7
132	88	Synaptotagmin1/2	1D12	mono	MBL	D156-3	11		AB_592780		22	14	8
133	17	Synaptotagmin2 (Rat)	8G2b	mono	MBL	D158-3		rat	AB_592781		21	12	7
134	131	T Cell Marker	KEN-5	mono	Santa Cruz	sc-59373	J2116	rabbit	AB_785902		18	13	6
135	143	T-bet	4B10	mono	Santa Cruz	sc-21749	H2317	human,mouse,rat	AB_628331		21	21	17
136	155	TERT	A-6	mono	Santa Cruz	sc-393013	K0218	human,mouse,rat			19	16	5
137	167	TERT	C-12	mono	Santa Cruz	sc-377511	F2518	human,mouse,rat	AB_11150127		21	15	6
138	108	TH	F-11	mono	Santa Cruz	sc-25269	C2118	human,mouse,rat	AB_628422		20	9	5
139	179	TH	A-1	mono	Santa Cruz	sc-374047	A2318	human,mouse,rat	AB_10918377		20	16	7
140	86	TPH2	CL2990	mono	Sigma	AMAb91108		human,mouse,rat	AB_2665804		22	21	13
141	56	TUBG2 (Mouse)		mono	MBL	D290-3	1	mouse	D290-3		22	19	10
142	120	Tyrosinase	T311	mono	Santa Cruz	sc-20035	K0317	human	AB_628420		20	22	16
143	14	Tyrosine hydroxylase	M-20	mono	Santa Cruz	sc-25269	H2510	human,mouse,rat	AB_628422		20	21	11
144	26	Tyrosine hydroxylase	EP1532Y	mono	Abcam	ab137869	GR3219792-2	human,mouse,rat	AB_2801410		22	12	8
145	33	Tyrosine Hydroxylase	EP1532Y	mono	abcam	ab137869	GR3219792-3	human,mouse,rat	AB_2801410		21	20	17
146	81	V5	V5-10	mono	sigma	V8012	128K4835	mouse	AB_261888		20	16	10

147	85	VIP		poly	Immunostar	20077	1339001	pig	AB_572270		19	18	10
148	132	α-synuclein	211	mono	Santa Cruz	sc-12767	K0218	human	:AB_628318		22	18	9
149	144	α-synuclein	2B2D1	mono	Santa Cruz	sc-53955	D0618	human,mouse,rat	AB_831743		19	12	5
150	156	α-synuclein	3H2897	mono	Santa Cruz	sc-69977	C0119	human,mouse,rat	AB_1118910		18	15	6
151	168	α-synuclein	LB 509	mono	Santa Cruz	sc-58480	J1917	human	AB_785898		18	17	7
152	180	α-synuclein	Syn 204	mono	Santa Cruz	sc-32280	J0617	human	AB_628319		19	21	11
153	93	α-Tubulin	B-5-1-2	mono	sigma	T6199	124KA4876	human,mouse,rat	AB_477583		17	15	7
154	58	c-fos poly		poly	abcam	ab190289	GR3253255-1		AB_2737414		17	16	9
155	70	c-fos poly		poly	abcam	ab190289	GR3313102-1		AB_2737414		16	16	7
156	82	c-fos poly		poly	abcam	ab209794	GR3198011-8		AB_2905616		23	16	9
157	94	c-fos poly		poly	abcam	ab209794	GR3266315-7		AB_2905616		22	18	9
158	23	Phospho-CaMKII (Thr286)	D21E4	mono	CST	Cat# 12716,	Lot 3		AB_2713889		23	18	13
159	35	CaMKII alpha (phospho Thr305)		poly	genetex	Cat# GTX 52345	Lot 821602176				21	20	15
160	47	CaMKII (Alpha-Specific) Antibody	6G9	mono	StressMarq-Biosciences	Cat# SMC-124D	Lot# 1011		AB_2275062		21	15	5
161	59	CaMKII beta	CB-beta-1	mono	invitrogen	Cat# 13-9800	Lot SA246802		AB_86565		22	14	5
162	71	Anti-Iba1 antibody [EPR16588]	EPR16588	mono	abcam	ab178846			AB_2636859		23	17	12
163	83	Anti-Iba1 antibody [EPR16589]	EPR16589	mono	abcam	ab178847			AB_2832244		22	10	5
164	95	Allograft inflammatory factor 1	HL22	mono	genetex	GTX635363			AB_2888516		20	19	15
165	12	Allograft inflammatory factor 1		poly	wako	019-19741	180201-4-1		AB_839504		21	20	12
166	24	Allograft inflammatory factor 1		poly	wako	019-19741	180319-1-1		AB_839504		20	19	12
167	36	Allograft inflammatory factor 1		poly	wako	019-19741	180425-2-1		AB_839504		22	19	10
168	48	Allograft inflammatory factor 1		poly	wako	019-19741	200520IBAR		AB_839504		22	18	7
169	60	Allograft inflammatory factor 1		poly	wako	019-19741	200521IBAR		AB_839504		21	19	15
170	72	Allograft inflammatory factor 1		poly	wako	019-19741	AKM6526		AB_839504		24	18	6
171	11	c-fos mono	2H2	mono	abcam	ab208942			AB_2747772		16	17	10
172	97	c-fos		poly	abcam	ab190289	GR3253255-1	human	AB_2737414		16	18	11
173	109	c-fos		poly	abcam	ab190289	GR3253255-1	human	AB_2737414		17	17	9
174	121	c-fos		poly	abcam	ab190289	GR3313102-1	human	AB_2737414		17	17	9
175	133	c-fos		poly	abcam	ab190289	GR3313102-1	human	AB_2737414		20	18	11
176	145	c-fos		poly	abcam	ab209794	GR3198011-8	human	AB_2905616		19	19	12
177	157	c-fos		poly	abcam	ab209794	GR3198011-8	human	AB_2905616		21	19	14
178	169	c-fos		poly	abcam	ab209794	GR3266315-7	human	AB_2905616		19	21	14
179	181	c-fos		poly	abcam	ab209794	GR3266315-7	human	AB_2905616		21	18	13
180	98	c-fos		poly	sigma	HPA018531	E106326	human	AB_1846576		22	19	13
181	110	c-fos		poly	sigma	HPA018531	E106326	human	AB_1846576		22	19	11
182	122	c-fos		poly	Santa-cruz	sc-52G	K1314	human	AB_2629503		22	20	10
183	134	c-fos		poly	Santa-cruz	sc-52G	K1314	human	AB_2629503		22	21	11
184	146	c-fos		poly	Santa-cruz	sc-52G	K1314	human	AB_2629503		20	19	11
185	158	c-fos	2H2	mono	abcam	ab208942	GR3264447-5	human,mouse,rat	AB_2747772		21	18	11
186	170	c-fos	2H2	mono	abcam	ab208942	GR3264447-5	human,mouse,rat	AB_2747772		20	18	10
187	182	c-fos	2H2	mono	abcam	ab208942	GR3264447-5	human,mouse,rat	AB_2747772		18	14	7
188	99	c-fos	9F6	mono	CST	2250S	9	human,mouse,rat	AB_2247211		18	15	7
189	111	c-fos	9F6	mono	CST	2250S	9	human,mouse,rat	AB_2247211		17	16	7
190	123	c-fos	9F6	mono	CST	2250S	9	human,mouse,rat	AB_2247211		21	22	12
191	135	c-fos	C-10	mono	Santa Cruz	sc-271243	F2119	human	AB_10610067		21	21	12
192	147	c-fos	C-10	mono	Santa Cruz	sc-271243	F2119	human	AB_10610067		21	20	13
193	159	c-fos	C-10	mono	Santa Cruz	sc-271243	F2119	human	AB_10610067		22	17	7
194	171	c-fos	D-1	mono	Santa Cruz	sc-8047	C2019	human	AB_627253		20	18	8
195	183	c-fos	D-1	mono	Santa Cruz	sc-8047	C2019	human	AB_627253		21	17	7

196	100	c-fos	D-1	mono	Santa Cruz	sc-8047	C2019	human	AB_627253	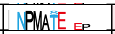	22	19	16
197	112	c-fos	E-8	mono	Santa Cruz	sc-166940	G1119	human	AB_10609634	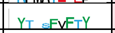	21	20	16
198	124	c-fos	E-8	mono	Santa Cruz	sc-166940	G1119	human	AB_10609634	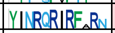	19	22	17
199	136	c-fos	E-8	mono	Santa Cruz	sc-166940	G1119	human	AB_10609634	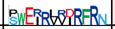	21	22	12
200	148	c-fos	E-8	mono	Santa Cruz	sc-166940	A2321	human	AB_10609634	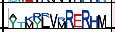	22	19	11
201	172	c-fos	E-8	mono	Santa Cruz	sc-166940	A2321	human	AB_10609634	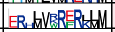	22	19	12
202	101	p53	Bp53-12	mono	Santa Cruz	sc-263	E2118	human,mouse,rat	AB_628084	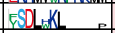	20	17	7
203	113	p53	Bp53-12	mono	Santa Cruz	sc-263	E2118	human,mouse,rat	AB_628084	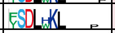	21	18	7
204	125	p53	C-11	mono	Santa Cruz	sc-55476	B0119	human,mouse,rat	AB_630061	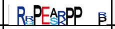	23	20	15
205	137	p53	C-11	mono	Santa Cruz	sc-55476	B0119	human,mouse,rat	AB_630061	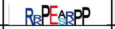	22	20	16
206	149	p53	D-11	mono	Santa Cruz	sc-17846	L1417	human,mouse,rat	AB_628081	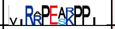	22	21	16
207	161	p53	D-11	mono	Santa Cruz	sc-17846	L1417	human,mouse,rat	AB_628081	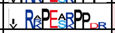	21	22	16
208	173	p53	DO-1	mono	Santa Cruz	sc-126	C1419	human,mouse,rat	AB_628082	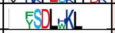	22	17	7
209	185	p53	DO-1	mono	Santa Cruz	sc-126	C1419	human,mouse,rat	AB_628082	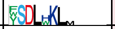	21	18	8
210	102	p53	DO-2	mono	Santa Cruz	sc-53394	J0617	human,mouse,rat	AB_785038	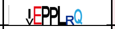	21	18	10
211	114	p53	DO-2	mono	Santa Cruz	sc-53394	J0617	human,mouse,rat	AB_785038	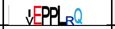	22	19	10
212	126	p53	DO-7	mono	Santa Cruz	sc-47698	B2019	human	AB_628083	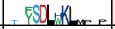	21	18	7
213	138	p53	DO-7	mono	Santa Cruz	sc-47698	B2019	human	AB_628083	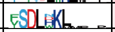	22	17	7
214	150	p53	Pab 1801	mono	Santa Cruz	sc-98	K1218	human,mouse,rat	AB_628085	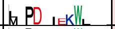	20	20	9
215	162	p53	Pab 1801	mono	Santa Cruz	sc-98	K1218	human,mouse,rat	AB_628085	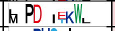	19	22	9
216	174	p53	Pab 240	mono	Santa Cruz	sc-99	K2817	human,mouse,rat	AB_628086	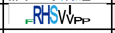	21	14	8
217	186	p53	Pab 240	mono	Santa Cruz	sc-99	K2817	human,mouse,rat	AB_628086	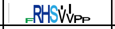	21	15	8
218	160	c-fos	E-8	mono	Santa Cruz	sc-166940	A2321	human	AB_10609634	N.D.	22	22	13
219	109	AFP	C3	mono	Santa Cruz	sc-8399	K0117	human,mouse,rat	AB_626665	N.D.	20	16	6
220	145	CA19-9	C4	mono	Santa Cruz	sc-517448	C2917			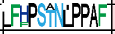	20	22	10
221	38	Dat		poly	Sigma	HPA013602	A97032	human	AB_1847484	N.D.	21	19	10
222	173	GFAP	GA-5	mono	Santa Cruz	sc-58766	B0119	human,mouse,rat	AB_783554	N.D.	20	21	18
223	43	Histone H4	D2X4V	mono	CST	14149	1	human		N.D.	19	16	8
224	114	IgA	A-9	mono	Santa Cruz	sc-373823	J2717	human	AB_11012153	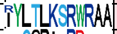	21	21	16
225	126	IgA	B-12	mono	Santa Cruz	sc-16634	J2017		AB_2093927	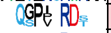	18	21	15
226	103	Macrophage Marker	MAC387	mono	Santa Cruz	sc-66204	L0718	human,mouse,rat	AB_2184280	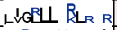	22	20	14
227	115	Melanoma Marker	HMB45	mono	Santa Cruz	sc-59305	B0618	human,mouse,rat	AB_627429	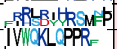	21	19	8
228	163	MHC class II	IBL-5/22	mono	Santa Cruz	sc-59322	I1918	mouse	AB_831551	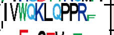	20	22	17
229	51	PGP9.5	EPR4118	mono	Abcam	ab108986	GR3183936-1	human,mouse,rat	AB_10891773	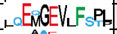	21	18	8
230	107	Rev-erbβ	D-8	mono	Santa Cruz	sc-398252	D1718	mosue		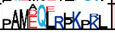	20	22	15
