# Supplementary material for: DECODE enables high-throughput mapping of antibody epitopes at single amino acid resolution
Source: PLoS Biol. 2025 Jan 23;23(1):e3002707. doi: 10.1371/journal.pbio.3002707 (PMC11756784; doi:10.1371/journal.pbio.3002707)
Supplement: S2 Table — (PDF) [file pbio.3002707.s015.pdf]

S2 Table. List of nucleic acid sequences used for peptide selection

Name	direction	Primer sequence
Primer 1	forward	CCTAATACGACTCACTATAGGGTTAACTTTAAGAAGGAGATATACATATG
Primer 2 (antigen)	reverse	GGTCGGCGGATCAAAGTAG
Primer 2 (antigen_OMe)	reverse	ggTCGGCGGATCAAAGTAG
Primer 2 (ver.2)	reverse	GGTCGGCGGATCAAAGTAGCTGCCGCTGCCGCTGCCGCA
DNA-PEG-CCPu		CTCCCGCCCCCCGTCC [SpC18]5CC[Pmycn]
Splint_1		GGGCGGGAGGGTCGGCGGATCAA
Library Template DNA sequence	(125 bp)	CCTAATACGACTCACTATAGGGTTAACTTTAAGAAGGAGATATACATATG<br>(NNK)nTGCGGCAGCGGCAGCGGCAGCTACTTTGATCCGCCGACC<br>(n = 12,N; A, T, G, C, K; T, G)
The Template mc1' DNA sequence	(140 bp)	CCTAATACGACTCACTATAGGGTTAACTTTAAGAAGGAGATATACATATGA<br>AGTACTCCCCAACCGACTGCAAGAAGGACTACAAGGACGACGACGACAAGT<br>GCGGCAGCGGCAGCGGCAGCTAGGACGGGGGGCGGAAA
