# Supplementary material for: DECODE enables high-throughput mapping of antibody epitopes at single amino acid resolution
Source: PLoS Biol. 2025 Jan 23;23(1):e3002707. doi: 10.1371/journal.pbio.3002707 (PMC11756784; doi:10.1371/journal.pbio.3002707)
Supplement: S3 Table — (PDF) [file pbio.3002707.s016.pdf]

S3 Table. List of barcode primer sequences used for NGS

Primer Name	Sequence	Primer Name	Sequence
Hiseq_F-001	ACGAGTGCGGTTAACTTTAAGAAGGAGATATACATATG	Hiseq_R-001	ACGAGTGCGCGCTGCCGCTGCCGCA
Hiseq_F-002	TCGCTCGACGTTAACTTTAAGAAGGAGATATACATATG	Hiseq_R-002	GCGCTCGACCGCTGCCGCTGCCGCA
Hiseq_F-003	CGACGCACTGTTAACTTTAAGAAGGAGATATACATATG	Hiseq_R-003	AGACGCACTCGCTGCCGCTGCCGCA
Hiseq_F-004	TGCACTGTAGTTAACTTTAAGAAGGAGATATACATATG	Hiseq_R-004	GGCACTGTACGCTGCCGCTGCCGCA
Hiseq_F-005	ATCAGACACGTTAACTTTAAGAAGGAGATATACATATG	Hiseq_R-005	CTCAGACACCGCTGCCGCTGCCGCA
Hiseq_F-006	GTATCGCGAGTTAACTTTAAGAAGGAGATATACATATG	Hiseq_R-006	TTATCGCGACGCTGCCGCTGCCGCA
Hiseq_F-007	CGTGTCTCTGTTAACTTTAAGAAGGAGATATACATATG	Hiseq_R-007	AGTGTCTCTCGCTGCCGCTGCCGCA
Hiseq_F-008	TTCGCGTGTGTTAACTTTAAGAAGGAGATATACATATG	Hiseq_R-008	GTCGCGTGTCGCTGCCGCTGCCGCA
Hiseq_F-009	TCTCTATGCGTTAACTTTAAGAAGGAGATATACATATG	Hiseq_R-009	CCTCTATGCCGCTGCCGCTGCCGCA
Hiseq_F-010	AGATACGTCGTTAACTTTAAGAAGGAGATATACATATG	Hiseq_R-010	TGATACGTCCGCTGCCGCTGCCGCA
Hiseq_F-011	GATAGTAGTGTTAACTTTAAGAAGGAGATATACATATG	Hiseq_R-011	TATAGTAGTCGCTGCCGCTGCCGCA
Hiseq_F-012	CGAGAGATAGTTAACTTTAAGAAGGAGATATACATATG	Hiseq_R-012	AGAGAGATACGCTGCCGCTGCCGCA
Hiseq_F-013	TTACGACGTGTTAACTTTAAGAAGGAGATATACATATG	Hiseq_R-013	GTACGACGTCGCTGCCGCTGCCGCA
Hiseq_F-014	ACACGTACTGTTAACTTTAAGAAGGAGATATACATATG	Hiseq_R-014	CCACGTACTCGCTGCCGCTGCCGCA
Hiseq_F-015	GGTCTAGTAGTTAACTTTAAGAAGGAGATATACATATG	Hiseq_R-015	TGTCTAGTACGCTGCCGCTGCCGCA
Hiseq_F-016	CCTACGTAGGTTAACTTTAAGAAGGAGATATACATATG	Hiseq_R-016	ACTACGTAGCGCTGCCGCTGCCGCA
Hiseq_F-017	TGTACTACTGTTAACTTTAAGAAGGAGATATACATATG	Hiseq_R-017	GGTACTACTCGCTGCCGCTGCCGCA
Hiseq_F-018	TCGACTACAGTTAACTTTAAGAAGGAGATATACATATG	Hiseq_R-018	CCGACTACACGCTGCCGCTGCCGCA
Hiseq_F-019	AGTAGACTAGTTAACTTTAAGAAGGAGATATACATATG	Hiseq_R-019	TGTAGACTACGCTGCCGCTGCCGCA
Hiseq_F-020	GACGAGTATGTTAACTTTAAGAAGGAGATATACATATG	Hiseq_R-020	TACGAGTATCGCTGCCGCTGCCGCA
Hiseq_F-021	CACTCTCGTGTTAACTTTAAGAAGGAGATATACATATG	Hiseq_R-021	AACTCTCGTCGCTGCCGCTGCCGCA
Hiseq_F-022	TAGAGACGAGTTAACTTTAAGAAGGAGATATACATATG	Hiseq_R-022	TAGAGACGACGCTGCCGCTGCCGCA
Hiseq_F-023	ACGTCGCTGTTAACTTTAAGAAGGAGATATACATATG	Hiseq_R-023	CCGTCGCTCGCTGCCGCTGCCGCA
Hiseq_F-024	GCATACGCGTTAACTTTAAGAAGGAGATATACATATG	Hiseq_R-024	TCATACGCCGCTGCCGCTGCCGCA
Hiseq_F-025	CCGCGAGTGTTAACTTTAAGAAGGAGATATACATATG	Hiseq_R-025	ACGCGAGTCGCTGCCGCTGCCGCA
Hiseq_F-026	TCTACTATGTTAACTTTAAGAAGGAGATATACATATG	Hiseq_R-026	GCTACTATCGCTGCCGCTGCCGCA
Hiseq_F-027	TCTGTACAGTTAACTTTAAGAAGGAGATATACATATG	Hiseq_R-027	CCTGTACACGCTGCCGCTGCCGCA
Hiseq_F-028	AGACTATAGTTAACTTTAAGAAGGAGATATACATATG	Hiseq_R-028	TGACTATACGCTGCCGCTGCCGCA
Hiseq_F-029	GGCGTCGTGTTAACTTTAAGAAGGAGATATACATATG	Hiseq_R-029	TGCGTCGTCGCTGCCGCTGCCGCA
Hiseq_F-030	CGTACGCCGTTAACTTTAAGAAGGAGATATACATATG	Hiseq_R-030	AGTACGCCCGCTGCCGCTGCCGCA
Hiseq_F-031	TTAGAAATGTTAACTTTAAGAAGGAGATATACATATG	Hiseq_R-031	GTAGAAATCGCTGCCGCTGCCGCA
Hiseq_F-032	AACGCTACGTTAACTTTAAGAAGGAGATATACATATG	Hiseq_R-032	CACGCTACCGCTGCCGCTGCCGCA
Hiseq_F-033	GAGTAGACGTTAACTTTAAGAAGGAGATATACATATG	Hiseq_R-033	TAGTAGACCGCTGCCGCTGCCGCA
Hiseq_F-034	CGACGTGAGTTAACTTTAAGAAGGAGATATACATATG	Hiseq_R-034	AGACGTGACGCTGCCGCTGCCGCA
Hiseq_F-035	TACACACAGTTAACTTTAAGAAGGAGATATACATATG	Hiseq_R-035	GACACACACGCTGCCGCTGCCGCA
Hiseq_F-036	AACACGTGGTTAACTTTAAGAAGGAGATATACATATG	Hiseq_R-036	CACACGTGCGCTGCCGCTGCCGCA
Hiseq_F-037	AACAGATCGTTAACTTTAAGAAGGAGATATACATATG	Hiseq_R-037	TACAGATCCGCTGCCGCTGCCGCA
Hiseq_F-038	GACGCTGTGTTAACTTTAAGAAGGAGATATACATATG	Hiseq_R-038	AACGCTGTCGCTGCCGCTGCCGCA
Hiseq_F-039	CAGTGTAGGTTAACTTTAAGAAGGAGATATACATATG	Hiseq_R-039	AAGTGTAGCGCTGCCGCTGCCGCA
Hiseq_F-040	TCGATCACGTTAACTTTAAGAAGGAGATATACATATG	Hiseq_R-040	GCGATCACCGCTGCCGCTGCCGCA
Hiseq_F-041	ACGCACTAGTTAACTTTAAGAAGGAGATATACATATG	Hiseq_R-041	CCGCACTACGCTGCCGCTGCCGCA
Hiseq_F-042	GCTAGCGAGTTAACTTTAAGAAGGAGATATACATATG	Hiseq_R-042	TCTAGCGACGCTGCCGCTGCCGCA
Hiseq_F-043	CCTATACAGTTAACTTTAAGAAGGAGATATACATATG	Hiseq_R-043	ACTATACACGCTGCCGCTGCCGCA
Hiseq_F-044	TGACGTAAGTTAACTTTAAGAAGGAGATATACATATG	Hiseq_R-044	GGACGTAACGCTGCCGCTGCCGCA
Hiseq_F-045	AGTGAGTAGTTAACTTTAAGAAGGAGATATACATATG	Hiseq_R-045	CGTGAGTACGCTGCCGCTGCCGCA
Hiseq_F-046	ACAGTATAGTTAACTTTAAGAAGGAGATATACATATG	Hiseq_R-046	TCAGTATACGCTGCCGCTGCCGCA
Hiseq_F-047	GCGCGATCGTTAACTTTAAGAAGGAGATATACATATG	Hiseq_R-047	ACGCGATCCGCTGCCGCTGCCGCA
Hiseq_F-048	CCTAGCAGTTAACTTTAAGAAGGAGATATACATATG	Hiseq_R-048	ACTAGCACGCTGCCGCTGCCGCA
Hiseq_F-049	TGCTCACGTTAACTTTAAGAAGGAGATATACATATG	Hiseq_R-049	TGCTCACCGCTGCCGCTGCCGCA
Hiseq_F-050	AGTATACGTTAACTTTAAGAAGGAGATATACATATG	Hiseq_R-050	CGTATACCGCTGCCGCTGCCGCA
Hiseq_F-051	AGTCAAGGTTAACTTTAAGAAGGAGATATACATATG	Hiseq_R-051	TGTCAAGCGCTGCCGCTGCCGCA
Hiseq_F-052	CAAGCTAGTTAACTTTAAGAAGGAGATATACATATG	Hiseq_R-052	AAAGCTACGCTGCCGCTGCCGCA
Hiseq_F-053	TGATCGTGTTAACTTTAAGAAGGAGATATACATATG	Hiseq_R-053	AGATCGTCGCTGCCGCTGCCGCA
Hiseq_F-054	AGCAGTAGTTAACTTTAAGAAGGAGATATACATATG	Hiseq_R-054	CGCAGTACGCTGCCGCTGCCGCA
Hiseq_F-055	AGCGTATGTTAACTTTAAGAAGGAGATATACATATG	Hiseq_R-055	TGCGTATCGCTGCCGCTGCCGCA
Hiseq_F-056	GGTACAGGTTAACTTTAAGAAGGAGATATACATATG	Hiseq_R-056	AGTACAGCGCTGCCGCTGCCGCA
Hiseq_F-057	CGTACTCGTTAACTTTAAGAAGGAGATATACATATG	Hiseq_R-057	AGTACTCCGCTGCCGCTGCCGCA
Hiseq_F-058	TTACGCTGTTAACTTTAAGAAGGAGATATACATATG	Hiseq_R-058	GTACGCTCGCTGCCGCTGCCGCA

Hiseq_F-059	ATATAGCGTTAACTTTAAGAAGGAGATATACATATG	Hiseq_R-059	CTATAGCCGCTGCCGCTGCCGCA
Hiseq_F-060	GACGTCAGTTAACTTTAAGAAGGAGATATACATATG	Hiseq_R-060	TACGTCACGCTGCCGCTGCCGCA
Hiseq_F-061	CAGTCGCGTTAACTTTAAGAAGGAGATATACATATG	Hiseq_R-061	AAGTCGCCGCTGCCGCTGCCGCA
Hiseq_F-062	TATATATGTTAACTTTAAGAAGGAGATATACATATG	Hiseq_R-062	GATATATCGCTGCCGCTGCCGCA
Hiseq_F-063	AATGCTAGTTAACTTTAAGAAGGAGATATACATATG	Hiseq_R-063	CATGCTACGCTGCCGCTGCCGCA
Hiseq_F-064	ACACGCGGTTAACTTTAAGAAGGAGATATACATATG	Hiseq_R-064	TCACGCGCGCTGCCGCTGCCGCA
Hiseq_F-065	GCGATAGGTTAACTTTAAGAAGGAGATATACATATG	Hiseq_R-065	ACGATAGCGCTGCCGCTGCCGCA
Hiseq_F-066	CCGCTGCGTTAACTTTAAGAAGGAGATATACATATG	Hiseq_R-066	ACGCTGCCGCTGCCGCTGCCGCA
Hiseq_F-067	TCTGACGGTTAACTTTAAGAAGGAGATATACATATG	Hiseq_R-067	GCTGACGCGCTGCCGCTGCCGCA
Hiseq_F-068	AGAGTCAGTTAACTTTAAGAAGGAGATATACATATG	Hiseq_R-068	CGAGTCACGCTGCCGCTGCCGCA
Hiseq_F-069	GGTAGTGGTTAACTTTAAGAAGGAGATATACATATG	Hiseq_R-069	TGTAGTGCGCTGCCGCTGCCGCA
Hiseq_F-070	CGTCACAGTTAACTTTAAGAAGGAGATATACATATG	Hiseq_R-070	AGTCACACGCTGCCGCTGCCGCA
Hiseq_F-071	TGTCGTCGTTAACTTTAAGAAGGAGATATACATATG	Hiseq_R-071	GGTCGTCCGCTGCCGCTGCCGCA
Hiseq_F-072	TACGTAGTTAACTTTAAGAAGGAGATATACATATG	Hiseq_R-072	CACGTACGCTGCCGCTGCCGCA
Hiseq_F-073	AACATGGTTAACTTTAAGAAGGAGATATACATATG	Hiseq_R-073	TACATGCGCTGCCGCTGCCGCA
Hiseq_F-074	GATTCTGTTAACTTTAAGAAGGAGATATACATATG	Hiseq_R-074	TATTCTCGCTGCCGCTGCCGCA
Hiseq_F-075	CAACATGTTAACTTTAAGAAGGAGATATACATATG	Hiseq_R-075	AAACATCGCTGCCGCTGCCGCA
Hiseq_F-076	TCAGTCGTTAACTTTAAGAAGGAGATATACATATG	Hiseq_R-076	TCAGTCCGCTGCCGCTGCCGCA
Hiseq_F-077	ACATGAGTTAACTTTAAGAAGGAGATATACATATG	Hiseq_R-077	CCATGACGCTGCCGCTGCCGCA
Hiseq_F-078	GCGACAGTTAACTTTAAGAAGGAGATATACATATG	Hiseq_R-078	TCGACACGCTGCCGCTGCCGCA
Hiseq_F-079	CCGTCTGTTAACTTTAAGAAGGAGATATACATATG	Hiseq_R-079	ACGTCTCGCTGCCGCTGCCGCA
Hiseq_F-080	TCTCATGTTAACTTTAAGAAGGAGATATACATATG	Hiseq_R-080	GCTCATCGCTGCCGCTGCCGCA
Hiseq_F-081	TCTCGCGTTAACTTTAAGAAGGAGATATACATATG	Hiseq_R-081	CCTCGCCGCTGCCGCTGCCGCA
Hiseq_F-082	AGACAAGTTAACTTTAAGAAGGAGATATACATATG	Hiseq_R-082	TGACAACGCTGCCGCTGCCGCA
Hiseq_F-083	TGAGATGTTAACTTTAAGAAGGAGATATACATATG	Hiseq_R-083	TGAGATCGCTGCCGCTGCCGCA
Hiseq_F-084	CGAGCGGTTAACTTTAAGAAGGAGATATACATATG	Hiseq_R-084	AGAGCGCGCTGCCGCTGCCGCA
Hiseq_F-085	TACGACGTTAACTTTAAGAAGGAGATATACATATG	Hiseq_R-085	GACGACCGCTGCCGCTGCCGCA
Hiseq_F-086	AGTAAAGTTAACTTTAAGAAGGAGATATACATATG	Hiseq_R-086	CGTAAACGCTGCCGCTGCCGCA
Hiseq_F-087	GGCTTGGTTAACTTTAAGAAGGAGATATACATATG	Hiseq_R-087	TGCTTGCGCTGCCGCTGCCGCA
Hiseq_F-088	CGTCGAGTTAACTTTAAGAAGGAGATATACATATG	Hiseq_R-088	AGTCGACGCTGCCGCTGCCGCA
Hiseq_F-089	TGTAACGTTAACTTTAAGAAGGAGATATACATATG	Hiseq_R-089	GGTAACCGCTGCCGCTGCCGCA
Hiseq_F-090	TGTGTAGTTAACTTTAAGAAGGAGATATACATATG	Hiseq_R-090	CGTGTACGCTGCCGCTGCCGCA
Hiseq_F-091	ATACGAGTTAACTTTAAGAAGGAGATATACATATG	Hiseq_R-091	TTACGACGCTGCCGCTGCCGCA
Hiseq_F-092	GTAGATGTTAACTTTAAGAAGGAGATATACATATG	Hiseq_R-092	TTAGATCGCTGCCGCTGCCGCA
Hiseq_F-093	CTGGTCGTTAACTTTAAGAAGGAGATATACATATG	Hiseq_R-093	ATGGTCCGCTGCCGCTGCCGCA
Hiseq_F-094	TTATAGGTTAACTTTAAGAAGGAGATATACATATG	Hiseq_R-094	TTATAGCGCTGCCGCTGCCGCA
Hiseq_F-095	ATCTACGTTAACTTTAAGAAGGAGATATACATATG	Hiseq_R-095	CTCTACCGCTGCCGCTGCCGCA
Hiseq_F-096	GTGTATGTTAACTTTAAGAAGGAGATATACATATG	Hiseq_R-096	TTGTATCGCTGCCGCTGCCGCA
Hiseq_F-097	AAGTGCGAGGTTAACTTTAAGAAGGAGATATACATATG	Hiseq_R-097	AAGTGCGAGCGCTGCCGCTGCCGCA
Hiseq_F-098	TAGAATCTAGTTAACTTTAAGAAGGAGATATACATATG	Hiseq_R-098	TAGAATCTACGCTGCCGCTGCCGCA
Hiseq_F-099	CGTAGATACGTTAACTTTAAGAAGGAGATATACATATG	Hiseq_R-099	CGTAGATACCGCTGCCGCTGCCGCA
Hiseq_F-100	TGATACACGGTTAACTTTAAGAAGGAGATATACATATG	Hiseq_R-100	TGATACACGCGCTGCCGCTGCCGCA
Hiseq_F-101	ACATGTTTCGTTAACTTTAAGAAGGAGATATACATATG	Hiseq_R-101	ACATGTTTCCGCTGCCGCTGCCGCA
Hiseq_F-102	GCTCAGAGTGTTAACTTTAAGAAGGAGATATACATATG	Hiseq_R-102	GCTCAGAGTCGCTGCCGCTGCCGCA
Hiseq_F-103	CGCGCACACGTTAACTTTAAGAAGGAGATATACATATG	Hiseq_R-103	CGCGCACACCGCTGCCGCTGCCGCA
Hiseq_F-104	TCAGTGCGCGTTAACTTTAAGAAGGAGATATACATATG	Hiseq_R-104	TCAGTGCGCCGCTGCCGCTGCCGCA
Hiseq_F-105	TACACTCGAGTTAACTTTAAGAAGGAGATATACATATG	Hiseq_R-105	TACACTCGACGCTGCCGCTGCCGCA
Hiseq_F-106	AGTCTAGCCGTTAACTTTAAGAAGGAGATATACATATG	Hiseq_R-106	AGTCTAGCCCGCTGCCGCTGCCGCA
Hiseq_F-107	GTCTGCAGGGTTAACTTTAAGAAGGAGATATACATATG	Hiseq_R-107	GTCTGCAGGCGCTGCCGCTGCCGCA
Hiseq_F-108	CGTGTGCCTGTTAACTTTAAGAAGGAGATATACATATG	Hiseq_R-108	CGTGTGCCTCGCTGCCGCTGCCGCA
Hiseq_F-109	TCTAGTAGCGTTAACTTTAAGAAGGAGATATACATATG	Hiseq_R-109	TCTAGTAGCCGCTGCCGCTGCCGCA
Hiseq_F-110	AATAGCTAGGTTAACTTTAAGAAGGAGATATACATATG	Hiseq_R-110	AATAGCTAGCGCTGCCGCTGCCGCA
Hiseq_F-111	GGCACTGCTGTTAACTTTAAGAAGGAGATATACATATG	Hiseq_R-111	GGCACTGCTCGCTGCCGCTGCCGCA
Hiseq_F-112	CACTAGCTGGTTAACTTTAAGAAGGAGATATACATATG	Hiseq_R-112	CACTAGCTGCGCTGCCGCTGCCGCA
Hiseq_F-113	TGCAACCACGTTAACTTTAAGAAGGAGATATACATATG	Hiseq_R-113	TGCAACCACCGCTGCCGCTGCCGCA
Hiseq_F-114	TAGTCGCTAGTTAACTTTAAGAAGGAGATATACATATG	Hiseq_R-114	TAGTCGCTACGCTGCCGCTGCCGCA
Hiseq_F-115	AGCTGATTTGTTAACTTTAAGAAGGAGATATACATATG	Hiseq_R-115	AGCTGATTTCGCTGCCGCTGCCGCA
Hiseq_F-116	GTAGTGCTCGTTAACTTTAAGAAGGAGATATACATATG	Hiseq_R-116	GTAGTGCTCCGCTGCCGCTGCCGCA
Hiseq_F-117	CTACACAGCGTTAACTTTAAGAAGGAGATATACATATG	Hiseq_R-117	CTACACAGCCGCTGCCGCTGCCGCA

Hiseq_F-118	TTGTGCGAAGTTAACTTTAAGAAGGAGATATACATATG	Hiseq_R-118	TTGTGCGAACGCTGCCGCTGCCGCA
Hiseq_F-119	AAGCAGACGTTAACTTTAAGAAGGAGATATACATATG	Hiseq_R-119	AAGCAGACCGCTGCCGCTGCCGCA
Hiseq_F-120	GATCTAGAGTTAACTTTAAGAAGGAGATATACATATG	Hiseq_R-120	GATCTAGACGCTGCCGCTGCCGCA
Hiseq_F-121	CAGAGTGCGTTAACTTTAAGAAGGAGATATACATATG	Hiseq_R-121	CAGAGTGCCGCTGCCGCTGCCGCA
Hiseq_F-122	TACTTATCGTTAACTTTAAGAAGGAGATATACATATG	Hiseq_R-122	TACTTATCCGCTGCCGCTGCCGCA
Hiseq_F-123	TACGCTATGTTAACTTTAAGAAGGAGATATACATATG	Hiseq_R-123	TACGCTATCGCTGCCGCTGCCGCA
Hiseq_F-124	AGTACTCTGTTAACTTTAAGAAGGAGATATACATATG	Hiseq_R-124	AGTACTCTCGCTGCCGCTGCCGCA
Hiseq_F-125	GGAGCAGCGTTAACTTTAAGAAGGAGATATACATATG	Hiseq_R-125	GGAGCAGCCGCTGCCGCTGCCGCA
Hiseq_F-126	CGATAGAAGTTAACTTTAAGAAGGAGATATACATATG	Hiseq_R-126	CGATAGAACGCTGCCGCTGCCGCA
Hiseq_F-127	TCTGTATCAGTTAACTTTAAGAAGGAGATATACATATG	Hiseq_R-127	TCTGTATCACGCTGCCGCTGCCGCA
Hiseq_F-128	ATAGAGACGTTAACTTTAAGAAGGAGATATACATATG	Hiseq_R-128	ATAGAGACCGCTGCCGCTGCCGCA
Hiseq_F-129	GTGCTAAAGTTAACTTTAAGAAGGAGATATACATATG	Hiseq_R-129	GTGCTAAACGCTGCCGCTGCCGCA
Hiseq_F-130	CGTAGCGTGTTAACTTTAAGAAGGAGATATACATATG	Hiseq_R-130	CGTAGCGTCGCTGCCGCTGCCGCA
Hiseq_F-131	TTATTGATGTTAACTTTAAGAAGGAGATATACATATG	Hiseq_R-131	TTATTGATCGCTGCCGCTGCCGCA
Hiseq_F-132	ATATAGCGGTTAACTTTAAGAAGGAGATATACATATG	Hiseq_R-132	ATATAGCGCGCTGCCGCTGCCGCA
Hiseq_F-133	ATAAGTCTGTTAACTTTAAGAAGGAGATATACATATG	Hiseq_R-133	ATAAGTCTCGCTGCCGCTGCCGCA
Hiseq_F-134	GTAGACGCGTTAACTTTAAGAAGGAGATATACATATG	Hiseq_R-134	GTAGACGCCGCTGCCGCTGCCGCA
Hiseq_F-135	CTGCGCTGGTTAACTTTAAGAAGGAGATATACATATG	Hiseq_R-135	CTGCGCTGCGCTGCCGCTGCCGCA
Hiseq_F-136	TAGTCATAGTTAACTTTAAGAAGGAGATATACATATG	Hiseq_R-136	TAGTCATACGCTGCCGCTGCCGCA
Hiseq_F-137	AAGATACAGTTAACTTTAAGAAGGAGATATACATATG	Hiseq_R-137	AAGATACACGCTGCCGCTGCCGCA
Hiseq_F-138	GACTGAGTGTTAACTTTAAGAAGGAGATATACATATG	Hiseq_R-138	GACTGAGTCGCTGCCGCTGCCGCA
Hiseq_F-139	CACTCTATGTTAACTTTAAGAAGGAGATATACATATG	Hiseq_R-139	CACTCTATCGCTGCCGCTGCCGCA
Hiseq_F-140	TGTAGCTTGTTAACTTTAAGAAGGAGATATACATATG	Hiseq_R-140	TGTAGCTTCGCTGCCGCTGCCGCA
Hiseq_F-141	AGCGAACTGTTAACTTTAAGAAGGAGATATACATATG	Hiseq_R-141	AGCGAACTCGCTGCCGCTGCCGCA
Hiseq_F-142	AATGCTCTGTTAACTTTAAGAAGGAGATATACATATG	Hiseq_R-142	AATGCTCTCGCTGCCGCTGCCGCA
Hiseq_F-143	GAGAATCGGTTAACTTTAAGAAGGAGATATACATATG	Hiseq_R-143	GAGAATCGCGCTGCCGCTGCCGCA
Hiseq_F-144	CACAGATGTTAACTTTAAGAAGGAGATATACATATG	Hiseq_R-144	CACAGATCGCTGCCGCTGCCGCA
Hiseq_F-145	TGACATAGTTAACTTTAAGAAGGAGATATACATATG	Hiseq_R-145	TGACATACGCTGCCGCTGCCGCA
Hiseq_F-146	AGCTCTGGTTAACTTTAAGAAGGAGATATACATATG	Hiseq_R-146	AGCTCTGCGCTGCCGCTGCCGCA
Hiseq_F-147	AGCATTGGTTAACTTTAAGAAGGAGATATACATATG	Hiseq_R-147	AGCATTGCGCTGCCGCTGCCGCA
Hiseq_F-148	CTTGACTGTTAACTTTAAGAAGGAGATATACATATG	Hiseq_R-148	CTTGACTCGCTGCCGCTGCCGCA
Hiseq_F-149	TGTCAGCGTTAACTTTAAGAAGGAGATATACATATG	Hiseq_R-149	TGTCAGCCGCTGCCGCTGCCGCA
Hiseq_F-150	ACATGCTGTTAACTTTAAGAAGGAGATATACATATG	Hiseq_R-150	ACATGCTCGCTGCCGCTGCCGCA
Hiseq_F-151	AGAACTCGTTAACTTTAAGAAGGAGATATACATATG	Hiseq_R-151	AGAACTCCGCTGCCGCTGCCGCA
Hiseq_F-152	GGTTACGGTTAACTTTAAGAAGGAGATATACATATG	Hiseq_R-152	GGTTACGCGCTGCCGCTGCCGCA
Hiseq_F-153	CGCTTAGGTTAACTTTAAGAAGGAGATATACATATG	Hiseq_R-153	CGCTTAGCGCTGCCGCTGCCGCA
Hiseq_F-154	TCGAAAGGTTAACTTTAAGAAGGAGATATACATATG	Hiseq_R-154	TCGAAAGCGCTGCCGCTGCCGCA
Hiseq_F-155	ACTCTGAGTTAACTTTAAGAAGGAGATATACATATG	Hiseq_R-155	ACTCTGACGCTGCCGCTGCCGCA
Hiseq_F-156	GTAGCAGAGTTAACTTTAAGAAGGAGATATACATATG	Hiseq_R-156	GTAGCAGACGCTGCCGCTGCCGCA
Hiseq_F-157	CTTAAGAGTTAACTTTAAGAAGGAGATATACATATG	Hiseq_R-157	CTTAAGACGCTGCCGCTGCCGCA
Hiseq_F-158	TACACTCGTTAACTTTAAGAAGGAGATATACATATG	Hiseq_R-158	TACACTCCGCTGCCGCTGCCGCA
Hiseq_F-159	ATCGACTGTTAACTTTAAGAAGGAGATATACATATG	Hiseq_R-159	ATCGACTCGCTGCCGCTGCCGCA
Hiseq_F-160	AATAGAGGTTAACTTTAAGAAGGAGATATACATATG	Hiseq_R-160	AATAGAGCGCTGCCGCTGCCGCA
Hiseq_F-161	GAGTCTGGTTAACTTTAAGAAGGAGATATACATATG	Hiseq_R-161	GAGTCTGCGCTGCCGCTGCCGCA
Hiseq_F-162	CAGACCAGTTAACTTTAAGAAGGAGATATACATATG	Hiseq_R-162	CAGACCACGCTGCCGCTGCCGCA
Hiseq_F-163	TACTTAAGTTAACTTTAAGAAGGAGATATACATATG	Hiseq_R-163	TACTTAACGCTGCCGCTGCCGCA
Hiseq_F-164	AGTGCATGTTAACTTTAAGAAGGAGATATACATATG	Hiseq_R-164	AGTGCATCGCTGCCGCTGCCGCA
Hiseq_F-165	GGCTGCGGTTAACTTTAAGAAGGAGATATACATATG	Hiseq_R-165	GGCTGCGCGCTGCCGCTGCCGCA
Hiseq_F-166	CGCATATGTTAACTTTAAGAAGGAGATATACATATG	Hiseq_R-166	CGCATATCGCTGCCGCTGCCGCA
Hiseq_F-167	TGCAGCAGTTAACTTTAAGAAGGAGATATACATATG	Hiseq_R-167	TGCAGCACGCTGCCGCTGCCGCA
Hiseq_F-168	TTAGCTGTTAACTTTAAGAAGGAGATATACATATG	Hiseq_R-168	TTAGCTCGCTGCCGCTGCCGCA
Hiseq_F-169	ATATCCGTTAACTTTAAGAAGGAGATATACATATG	Hiseq_R-169	ATATCCCGCTGCCGCTGCCGCA
Hiseq_F-170	GTCCACGTTAACTTTAAGAAGGAGATATACATATG	Hiseq_R-170	GTCCACCGCTGCCGCTGCCGCA
Hiseq_F-171	CTAAACGTTAACTTTAAGAAGGAGATATACATATG	Hiseq_R-171	CTAAACCGCTGCCGCTGCCGCA
Hiseq_F-172	TATGCAAGTTAACTTTAAGAAGGAGATATACATATG	Hiseq_R-172	TATGCAACGCTGCCGCTGCCGCA
Hiseq_F-173	AATTTTGTTAACTTTAAGAAGGAGATATACATATG	Hiseq_R-173	AATTTTCGCTGCCGCTGCCGCA
Hiseq_F-174	GCGTTCGTTAACTTTAAGAAGGAGATATACATATG	Hiseq_R-174	GCGTTCCGCTGCCGCTGCCGCA
Hiseq_F-175	CAGCACGTTAACTTTAAGAAGGAGATATACATATG	Hiseq_R-175	CAGCACCGCTGCCGCTGCCGCA
Hiseq_F-176	TACAACCGTTAACTTTAAGAAGGAGATATACATATG	Hiseq_R-176	TACAACCCGCTGCCGCTGCCGCA

Hiseq_F-177	TACAGATAGTTAACTTTAAGAAGGAGATATACATATG	Hiseq_R-177	TACAGATACGCTGCCGCTGCCGCA
Hiseq_F-178	AGTGTTGTTAACTTTAAGAAGGAGATATACATATG	Hiseq_R-178	AGTGTTCGCTGCCGCTGCCGCA
Hiseq_F-179	TGTGTGGGTTAACTTTAAGAAGGAGATATACATATG	Hiseq_R-179	TGTGTGGCGCTGCCGCTGCCGCA
Hiseq_F-180	CGTGAGGTTAACTTTAAGAAGGAGATATACATATG	Hiseq_R-180	CGTGAGCGCTGCCGCTGCCGCA
Hiseq_F-181	TTAGCCCGTTAACTTTAAGAAGGAGATATACATATG	Hiseq_R-181	TTAGCCCCGCTGCCGCTGCCGCA
Hiseq_F-182	AGCTTTGTTAACTTTAAGAAGGAGATATACATATG	Hiseq_R-182	AGCTTTCGCTGCCGCTGCCGCA
Hiseq_F-183	GGACCGGTTAACTTTAAGAAGGAGATATACATATG	Hiseq_R-183	GGACCGCGCTGCCGCTGCCGCA
Hiseq_F-184	CCCAGTGTTAACTTTAAGAAGGAGATATACATATG	Hiseq_R-184	CCCAGTCGCTGCCGCTGCCGCA
Hiseq_F-185	TGCTTAGTTAACTTTAAGAAGGAGATATACATATG	Hiseq_R-185	TGCTTACGCTGCCGCTGCCGCA
Hiseq_F-186	AGCCCTGTTAACTTTAAGAAGGAGATATACATATG	Hiseq_R-186	AGCCCTCGCTGCCGCTGCCGCA
Hiseq_F-187	GCTTGATGTTAACTTTAAGAAGGAGATATACATATG	Hiseq_R-187	GCTTGATCGCTGCCGCTGCCGCA
Hiseq_F-188	GCTGTCGTTAACTTTAAGAAGGAGATATACATATG	Hiseq_R-188	GCTGTCCGCTGCCGCTGCCGCA
Hiseq_F-189	CCGGCAGTTAACTTTAAGAAGGAGATATACATATG	Hiseq_R-189	CCGGCACGCTGCCGCTGCCGCA
Hiseq_F-190	TCTGAGGTTAACTTTAAGAAGGAGATATACATATG	Hiseq_R-190	TCTGAGCGCTGCCGCTGCCGCA
Hiseq_F-191	ACCCTAGTTAACTTTAAGAAGGAGATATACATATG	Hiseq_R-191	ACCCTACGCTGCCGCTGCCGCA
Hiseq_F-192	GCACTCAGTTAACTTTAAGAAGGAGATATACATATG	Hiseq_R-192	GCACTCACGCTGCCGCTGCCGCA
