# Supplementary material for: DECODE enables high-throughput mapping of antibody epitopes at single amino acid resolution
Source: PLoS Biol. 2025 Jan 23;23(1):e3002707. doi: 10.1371/journal.pbio.3002707 (PMC11756784; doi:10.1371/journal.pbio.3002707)
Supplement: S4 Table — (PDF) [file pbio.3002707.s017.pdf]

S4 Table. List of synthetic peptide sequences used in ELISA

cross reactivity ELISA

clone	synthesized sequence	protein positions	mutation position
2H2	TEPFDDFLFPASSRPSGSET	266-285	WT
9F6	ASPAGDSLSYYHSPADSFSS	20-39	WT
C-10	KVEQLSPEEEEKRRIRRE	130-144	WT

hot-spot ELISA(c-fos)

clone	synthesized sequence	protein positions	mutation position
9F6	PAGDSLSYYHSPADS	21-35	WT
9F6	AAGDSLSYYHSPADS	21-34	P21A
9F6	PGGDSLSYYHSPADS	21-34	A22G
9F6	PAADSLSYYHSPADS	21-34	G23A
9F6	PAGASLSYYHSPADS	21-34	D24A
9F6	PAGDALSYYHSPADS	21-34	S25A
9F6	PAGDSASYYHSPADS	21-34	L26A
9F6	PAGDSLAYYHSPADS	21-34	S27A
9F6	PAGDSLSAYHSPADS	21-34	Y28A
9F6	PAGDSLSYAHSPADS	21-34	Y29A
9F6	PAGDSLSYYASPADS	21-34	H30A
9F6	PAGDSLSYYHAPADS	21-34	S31A
9F6	PAGDSLSYYHSAADS	21-34	P32A
9F6	PAGDSLSYYHSPGDS	21-34	A33A
9F6	PAGDSLSYYHSPAAS	21-34	D34A

hot-spot ELISA(c-fos)

clone	synthesized sequence	protein positions	mutation position
2H2	FDDFLFPASSRPSGS	268-282	
2H2	ADDFLFPASSRPSGS	268-282	F268A
2H2	FADFLFPASSRPSGS	268-282	D269A
2H2	FDAFLFPASSRPSGS	268-282	D270A
2H2	FDDALFPASSRPSGS	268-282	F271A
2H2	FDDFAFPASSRPSGS	268-282	L272A
2H2	FDDFLAPASSRPSGS	268-282	F273A
2H2	FDDFLFAASSRPSGS	268-282	P274A
2H2	FDDFLFPGSSRPSGS	268-282	A275G
2H2	FDDFLFPAASRPSGS	268-282	S276A
2H2	FDDFLFPASARPSGS	268-282	S277A
2H2	FDDFLFPASSAPSGS	268-282	R278A
2H2	FDDFLFPASSRASGS	268-282	P279A
2H2	FDDFLFPASSRPAGS	268-282	S280A
2H2	FDDFLFPASSRPSAS	268-282	G281A

hot-spot ELISA(c-fos)

clone	synthesized sequence	protein positions	mutation position
C-10	KVEQLSPEEEEKRRIRRE	130-144	
C-10	KVAQLSPEEEEKRRIRRE	130-144	E130A
C-10	KVEALSPEEEEKRRIRRE	130-144	Q131A
C-10	KVEQASPEEEEKRRIRRE	130-144	L132A
C-10	KVEQLAPEEEEKRRIRRE	130-144	S133A
C-10	KVEQLSAEEEEKRRIRRE	130-144	P134A
C-10	KVEQLSPAEEEKRRIRRE	130-144	E135A
C-10	KVEQLSPEAEEKRRIRRE	130-144	E136A
C-10	KVEQLSPEEAEKRRIRRE	130-144	E137A
C-10	KVEQLSPEEEAKRRIRRE	130-144	E138A
C-10	KVEQLSPEEEEARRIRRE	130-144	K139A
C-10	KVEQLSPEEEEKARIRRE	130-144	R140A
C-10	KVEQLSPEEEEKRAIRRE	130-144	R141A
C-10	KVEQLSPEEEEKRRARRE	130-144	I142A
C-10	KVEQLSPEEEEKRRIARE	130-144	R143A

cross reactivity ELISA

No.	gene	synthesized sequence	protein positions
pep1	AHNAK2	EVLTVESPEEEAMTKYSQESWF	5169-5190
pep2	LAMA1	LPPKSLSPESEWLVTFATTNSS	2499-2520
pep3	FOSL2	RRDEQLSPEEEEKRRIRRERNK	114-135
pep4	PLCD4	LCFRVLVPELAMLRFVVMDYDW	687-708
pep5	FOS	GKVEQLSPEEEEKRRIRRERNK	127-148

hot-spot ELISA(NeuN)

clone	synthesized sequence	protein positions	mutation position
A60	MAQPYPPAQYPPPPQ		WT
A60	AAQPYPPAQYPPPPQ	1-15	M1A
A60	MGQPYPPAQYPPPPQ	1-15	A2G
A60	MAAPYPPAQYPPPPQ	1-15	Q3A
A60	MAQAYPPAQYPPPPQ	1-15	P4A
A60	MAQPAPPAQYPPPPQ	1-15	Y5A
A60	MAQPYAPAQYPPPPQ	1-15	P6A
A60	MAQPYPAAQYPPPPQ	1-15	P7A
A60	MAQPYPPGQYPPPPQ	1-15	A8G
A60	MAQPYPPAAYPPPPQ	1-15	Q9A
A60	MAQPYPPAQAPPPPQ	1-15	Y10A
A60	MAQPYPPAQYAPPPQ	1-15	P11A
A60	MAQPYPPAQYPAPPQ	1-15	P12A
A60	MAQPYPPAQYPPAPQ	1-15	P13A
A60	MAQPYPPAQYPPPAQ	1-15	P14A

cross reactivity ELISA

clone	synthesized sequence	protein positions	mutation position
Bp53-12, DO-1, DO-7	SQETFSDLWKLLPENN	15-30	WT
Pab1801	DLMLSPDDIEQWFTEDP	42-58	WT
Pab240	DRNTFRHSVVVPYEP	208-222	WT

hot-spot ELISA

clone	synthesized sequence	protein positions	mutation position
Bp53-12, DO-1, DO-7	SQETFSDLWKLLPENN	15-30	WT
Bp53-12, DO-1, DO-7	SAETFSDLWKLLPENN	15-30	Q16A
Bp53-12, DO-1, DO-7	SQATFSDLWKLLPENN	15-30	E17A
Bp53-12, DO-1, DO-7	SQEAFSDLWKLLPENN	15-30	T18A
Bp53-12, DO-1, DO-7	SQETASDLWKLLPENN	15-30	F19A
Bp53-12, DO-1, DO-7	SQETFADLWKLLPENN	15-30	S20A
Bp53-12, DO-1, DO-7	SQETFSALWKLLPENN	15-30	D21A
Bp53-12, DO-1, DO-7	SQETFSDAWKLLPENN	15-30	L22A
Bp53-12, DO-1, DO-7	SQETFSDLAKLLPENN	15-30	W23A
Bp53-12, DO-1, DO-7	SQETFSDLWALLPENN	15-30	K24A
Bp53-12, DO-1, DO-7	SQETFSDLWKALPENN	15-30	L25A
Bp53-12, DO-1, DO-7	SQETFSDLWKLAPENN	15-30	L26A
Bp53-12, DO-1, DO-7	SQETFSDLWKLLAENN	15-30	P27A
Bp53-12, DO-1, DO-7	SQETFSDLWKLLPANN	15-30	E28A
Bp53-12, DO-1, DO-7	SQETFSDLWKLLPEAN	15-30	N29A

cross reactivity ELISA

cat#	lot.	synthesized sequence	protein positions
ab190289	GR3253255-1	EEAFTLPLLNDPEPKPSVE	236-255
ab190289	GR3253255-1	ELKTEPFDDFLFPASSRP	263-271
ab190289	GR3253255-1,GR3313102-1	FPASSRPSGSETARSVPDM	274-293
ab190289	GR3313102-1	RPACKIPDDLGFPEEMSV	201-219
ab190289	GR3313102-1	PSAGAYSRAGVVKTMTGG	101-119
ab209794	GR3198011-8, GR3266315-7	KTMTGGRAQSIGRRGKVE	113-131
ab209794	GR3198011-8	RGKVEQLSPEEEEKRR	126-142
ab209794	GR3198011-8	AAKCRNRRRELTDTLQA	151-168
ab209794	GR3266315-7	APSQTRAPHPFGVPAPS	86-103
ab209794	GR3266315-7	PEEEEKRRIRRERNKMAAA	134-153
