# Supplementary material for: DECODE enables high-throughput mapping of antibody epitopes at single amino acid resolution
Source: PLoS Biol. 2025 Jan 23;23(1):e3002707. doi: 10.1371/journal.pbio.3002707 (PMC11756784; doi:10.1371/journal.pbio.3002707)
Supplement: Attachment — Submitted filename: 241014_HS_English_Responses to Reviewers+Fig_re.pdf [file pbio.3002707.s021.pdf]

Responses to Reviewers' Concerns for Matsumoto and Harada et al., "High-throughput and Unbiased Peptide Analysis for Genome-wide Epitope Identification by Single Amino Acid Resolution".

We sincerely appreciate the constructive feedback provided by the two reviewers. We are truly grateful for the time and effort they invested, which has played a significant role in enhancing the quality of the manuscript. In response to the reviewers' concerns, we have revised the manuscript by adding new text and experiments to improve its rigor and clarity.

First, the primary update involves adding a discussion that clearly compares previous studies on antibody epitope analysis based on peptide selection with our developed DECODE method. This highlights the strength of the DECODE method, which, due to its foundation on the mRNA display method with the largest library size among peptide selections, can provide epitope information with very precise single amino acid resolution. This is expected to be advantageous in the epitope analysis of polyclonal antibodies and very diverse blood antibodies (Reviewer 1_Concern #1, Reviewer 2_Concern #1).

Secondly, we added a detailed description regarding the throughput of peptide selection using the DECODE method. We demonstrated through additional experiments that the improved ligation efficiency due to the 2'OMe modification, a key feature of the DECODE method, allows us to omit the electrophoretic purification step of ligation products, as required in conventional methods, while still achieving the same peptide convergence efficiency (Reviewer 2_Concern #2). Additionally, we showed that a single researcher manually performed three rounds of peptide selection on up to 96 antibody samples simultaneously over five days, converging sufficiently epitope candidate peptides as demonstrated by qPCR data (Reviewer 1_Concern #2, Reviewer 2_Concern #5). The throughput of the DECODE method is more than 100 times higher compared to conventional mRNA display methods or general peptide array and selection-based epitope screenings (Reviewer 2_Concern #3).

Thirdly, we performed additional ELISA experiments against antigens derived from other proteins to demonstrate the accuracy of the cross-reactivity predicted by the DECODE method. For the anti-c-fos antibody clone C-10, we found that similar epitope motifs existed on the sequences of four proteins that scored higher than the target protein. The ELISA results confirmed that the antibody indeed recognized these sequences (Reviewer 2_Concern #4).

Furthermore, we thoroughly discussed the limitations of the DECODE method in analyzing conformational epitopes and the specific considerations related to mRNA display. We defined conformational epitopes as structural epitopes composed of discontinuous amino acids that do not fit within a 12-residue range. We then discussed the range within which DECODE can analyze epitopes and how it can potentially address conformational epitopes in the future (Reviewer 1_Concern #5). Additionally, we described the measures taken in this study to prevent RNA degradation (Reviewer

1_Concern #1).

Lastly, we clarified ambiguous terms in the manuscript. Specifically, we explained the term 'identified epitope peptide' as a peptide sequence resulting from peptide selection, which includes the consecutive 12 amino acid sequence with the highest DECODE score on the target protein, along with a few flanking residues (Reviewer 1_Concern #7). Additionally, we revised the detailed notation based on the feedback reviewers provided (Reviewer 1_Concern #4, 6, 8, Reviewer 2_Concern #6-11).

With these revisions, it has become more evident that the DECODE method demonstrates sufficient throughput to achieve comprehensive epitope analysis, accurately identifies hotspot residues from monoclonal antibodies to diverse blood antibodies, and can estimate genome-wide cross-reactivity. This emphasizes the novelty of our research in simultaneously achieving these three advantages.

Response to Reviewer #1:

Major concerns:

concern #1

1. The review of the related studies/ technologies is not sufficient. Highly related methods, such as the phage display based strategy from Epitopic and the AbMap strategy have not been well discussed. A clear comparison among DECODE and these strategies is highly recommended, especially, a sdie-by-side comparison between DECODE and AbMap;

Thank you very much for your valuable suggestions to thoroughly compare the DECODE method with conventional peptide selection methods. I would like to review AbMap [Qi H, Ma M, Hu C, et.al. Mol Cell Proteomics. 2021;20:100059.], which is based on phage display, and IMUNE (later SERA) [Pantazes RJ, et.al. Sci Rep. 2016; 2;6:30312., Kamath, K., Reifert, J., Johnston, T. et al. Sci Rep 10, 5294 (2020)., Haynes, W.A., Kamath, K., Bozekowski, J. et al. Commun Biol 4, 1317 (2021).], which is based on bacterial display.

Firstly, we'd like to discuss the accuracy of epitope analysis. Focusing on the peptide library size, DECODE has a library size of 1.5x10^13, which is larger than AbMap (<10^9) and IMUNE (10^10). This larger library size enhances the accuracy of predicting the importance of epitope hotspot residues. This is particularly effective when targeting highly diverse blood antibodies, allowing for the prediction of epitope motifs at a single amino acid resolution. For example, in the epitope analysis of COVID-19 serum antibodies by AbMap, the diversity of blood antibodies is addressed by enriching and purifying antigen-specific antibodies using

Spike protein before performing peptide selection. However, this method depends on antigen proteins, making it difficult to explore unknown antigens. In contrast, DECODE has the advantage of detecting EAE-specific peptides while maintaining the diversity of serum antibodies without depending on antigen proteins. IMUNE also has the potential to explore unknown antigens by directly binding peptide libraries without reducing the diversity of blood antibodies. However, for single amino acid resolution epitope prediction, where the importance of hotspot residues and the possibility of amino acid substitution with similar side chain properties are considered, DECODE's larger library size is advantageous.

Next, comparing throughput, all three methods use 96-well plate-based peptide selection. AbMap conducts one round, IMUNE conducts two rounds, and DECODE conducts three rounds of peptide selection. According to the results of peptide enrichment, two rounds are sufficient for DECODE. Therefore, the throughput of peptide selection for all three methods is comparable. However, the DECODE method involves a series of operations such as transcription, translation, binding reaction, washing, reverse transcription, and peptide elution that are already carried out through simple pipetting, making it possible to improve throughput with automated liquid handlers. Additionally, while AbMap and IMUNE use bacterial-mediated translation reactions that result in larger reaction volumes, DECODE's reaction volume can accommodate 384-well plates, allowing for further parallelization.

Regarding binding reactions, AbMap (phage display) and IMUNE (cell surface display) have higher antigen density compared to DECODE, which expresses peptides as single molecules from single genes. In peptide selection for epitope analysis, the high binding affinity of the target antibody itself minimizes the effect of antigen density differences.

In terms of peptide analysis methods, AbMap and IMUNE use alignment and clustering, which can introduce bias. DECODE matches peptide sequences obtained via NGS to the Protein Data Base to predict genome-wide cross-reactivity, allowing for unbiased epitope identification. However, DECODE may miss sequences not present in the PDB.

In conclusion, DECODE utilizes its large library size to detect epitope motifs with high precision. Its throughput is comparable to AbMap and IMUNE but can be further enhanced with automated liquid handlers and 384-well plates. The DECODE method excels in predicting cross-reactivity on the PDB but has limitations in detecting out of database.

This discussion has been added on line 422 of page 18.

concern #2

2. It is not clear how many rounds of selection is recommend? If several rounds of selection is required, the samples that could be analyzed in one test should be very limited;

We appreciate your valuable comments on the optimal number of peptide selection rounds. In this study, we recommend three rounds of selection for commercial antibodies (monoclonal and polyclonal antibodies) and mouse serum, and have indeed conducted three rounds of selection. According to the NGS results, two rounds are sufficient for high-affinity commercial antibodies or highly diverse blood antibodies, where we do not want to over-concentrate based on binding affinity. We have added this detail on line 282 of page 12 and included a table summarizing the qPCR results of the three rounds in Table S1.

concern #3

3. The limitations of the DECODE strategy should be sufficiently discussed, such as additional consideration for preparing RNA.

Thank you for pointing out the need to discuss the limitations of RNA preparation in the DECODE method based on mRNA display. In the DECODE method, extreme caution is necessary to prevent the degradation of mRNA by RNase in order to retain the genetic information of the peptide library.

In this study, to avoid such degradation, we meticulously maintained our equipment with RNase Quiet (Nacalai), which removes nucleases and extra nucleic acids, and always used RNase-Free water for rinsing.

Additionally, regarding experimental conditions, we paid careful attention to pH and metal ion concentration, which affect RNA degradation, and avoided long-term incubation. Specifically, incubation under high metal ion concentration conditions leads to RNA degradation, so we quenched the reaction with EDTA in the shortest possible time. Where necessary, you can also incorporate the use of RNase inhibitors. By preventing the loss of the library through these measures, we were able to identify epitopes of polyclonal antibodies and serum antibodies. This discussion has been added on page 26, line 621.

Minor concerns:

concern #4

1. Line 47, "to demonstrated" should be "to demonstrate";

We have revised the sections highlighted with yellow markers as per your suggestions on line 47 of page 3.

concern #5

2. It is known that ~80% of the antibodies are recognizing conformational epitopes, especially the therapeutic antibodies. How to resolve the conformational epitopes? More discussion is

needed;

I really appreciate your comments on the very important point concerning the definition of conformational epitopes. Generally, conformational epitopes are defined as epitopes formed by amino acids that are far apart in the primary structure but come into contact in the three-dimensional tertiary structure. However, there is no specific definition for the number of residues that must be apart to be called a conformational epitope. In other words, recognized amino acids that are even slightly discontinuous may be classified as conformational epitopes.

Therefore, even though most antibodies are said to recognize conformational epitopes, if the peptides fall within the range of linear peptide selection, it is possible to identify epitopes consisting of discontinuous amino acids.

In this study, we define conformational epitopes as those composed of discontinuous amino acids that do not contain within a range of 12 residues. For antibodies recognizing such epitopes, it is challenging to construct all proteins with three-dimensional structures using the DECODE method with completely random sequences, so it is necessary to perform peptide selection using structural scaffolds with variable regions. Indeed, the PURE system allows the expression of scaffold proteins, constructing of several hundred amino acid residues with variable regions like scFv, in peptide selection [Ohashi H. et al. Biochem Biophys Res Commun. 2007 352(1):270-6.]. Additionally, chaperones can be added to the PURE system to facilitate the expression of proteins with three-dimensional structures. Thus, for conformational epitopes composed of amino acids separated by more than 12 residues, it is necessary to perform peptide selection using partially randomized scaffold protein libraries. This discussion has been added on page 20, line 481.

concern #6

3. Is GPU really necessary for the data analysis?

As mentioned in Concern #1, during the DECODE method, NGS involves deeply sequencing approximately 100,000 to 1 million reads per antibody sample and matching all obtained peptide sequences to the entire PDB sequence to calculate similarity, which results in a very large computational workload. This analysis is necessary to identify epitopes of antibodies with unknown antigen proteins, to check cross-reactivity to various proteins, and even inter-species cross-reactivity. Of course, while it is not impossible to perform this computation using a CPU with multithreading, the GPU would be faster. If the proteins to be analyzed are limited, CPU computation would be sufficient.

concern #7

4. Please clearly define what is the "identified epitope peptides";

We are grateful for your suggestion. The term 'identified epitope peptides' on line 232 refers to the peptide sequences converged by selection, which include the consecutive 12 amino acid sequence with the highest DECODE score on the target protein (c-fos), along with a few flanking residues. This definition has been supplemented on line 239 of page 11.

concern #8

5. Please add the scores directly to the logos.

We have added DECODE scores to all sequence logos present in the figures.

Response to Reviewer #2:

concern #1

p.7, lines 135-138: The authors state the shortcomings of "conventional mRNA display methods", but because protocols and formats for mRNA display vary among research groups, they should cite the literature for the conventional methods they describe here.

We are grateful for your valuable suggestion. The existing method referred to here basically points to the protocol of the mRNA display method developed by Roberts RW and colleagues in 1997 [Roberts RW, Szostak JW. Proc Natl Acad Sci U S A. 1997 Nov;94(23):12297–302.].

A noteworthy point about this basic protocol is that due to the nucleotide addition by T7 RNA polymerase, approximately 50% of by-products are generated during in vitro transcription, which hinders the linkage between mRNA and Puromycin DNA (Pu-DNA). Therefore, in the protocol, it is necessary to separate the mRNA-Pu-DNA linkage product by acrylamide gel electrophoresis, cut out the gel, purify it, and extract the product. This electrophoresis and gel cutting purification process is particularly difficult to perform in parallel for hundreds of samples, making epitope analysis targeting hundreds of antibodies unrealistic. Incidentally, even in recent years, gel electrophoresis purification of ligation products has been performed for mRNA display, as reported by Sabrina Galiñanes Reyes and colleagues in 2021 [Reyes SG, Kuruma Y, Fujimi M, Yamazaki M, Eto S, Nishikawa S, et al. Biotechnol Bioeng. 2021;118(4):1702–15.]. The citation numbers for these references are indicated on page 7, line 135.

The DECODE method succeeded in inhibiting nucleotide addition by T7 polymerase through 2'-OMe modification of template DNA, improving the mRNA-Pu-DNA linkage efficiency to 90%, and eliminating the electrophoresis and purification processes, thereby achieving throughput for hundreds of antibodies.

concern #2

p.8, lines 167-172: The authors show that using the improved mRNA display, the percentage of peptides that bind to the FLAG antibody exceeded 90% after three rounds, but they should show how much the enrichment efficiency improved compared to when the unmodified protocol was used.

Thank you for your comment on the very important point regarding peptide concentration efficiency. In this study, we optimized the buffer composition for all steps, including PCR, in vitro transcription, binding reactions, washing, and extraction, to improve the throughput of

mRNA display. Therefore, direct comparison with conventional methods is difficult. Instead, we focused on the most important improvement of our modified mRNA display, removing purification after ligation between Pu-DNA and mRNA library, and conducted additional experiments comparing the peptide concentration efficiency of the DECODE method with 2'-OMe modification and the conventional method without 2'-OMe modification, where the ligation products were purified using acrylamide gel.

As a result, the DECODE method with 2'-OMe modification showed peptide concentration efficiency comparable to that of the conventional method without 2'-OMe modification and with gel purification. This indicates that the DECODE method can achieve higher throughput than conventional methods without compromising peptide concentration efficiency, even without the gel purification step.

These results have been added to figure 1C and on page 8, line 170.

concern #3

p.8, lines 177-179: The authors stated that "Ultimately, it will be possible to perform peptide selection targeting at least approximately 100 individual antibodies per week using multiwell plates, increasing the throughput by more than 100 times using existing methods." When they actually performed the selection against the 230 antibodies shown in Table S1, did they not use 96-well microplates or an automatic pipetting machine? (This is not described in the Method section.) If the authors did it manually, how long did it take? (e.g., more than 230 weeks?)

We appreciate your guidance on the need to write more specific about throughput and parallelization. In this study, to perform epitope screening for 230 antibodies, we used 96-well PCR plates (Nippon Genetics*) for the steps up to PCR, in vitro transcription, ligation, and transcription. For the steps involving antibody bead binding, washing, and extraction, we used 96-Well U-Shaped-Bottom plates (Thermo Fisher Scientific**). All steps were manually performed using a multi-pipettor. The binding and washing steps for the antibody beads involved adding buffer, shaking with a shaker, and then removing the buffer.

In this study, it took one researcher five days to perform three rounds of peptide selection for 96 antibody samples using the above method, in compliance with labor standards. Therefore, it took approximately 15 days for one researcher to perform peptide selection for 230 samples. Excluding the DNA purification step after PCR, the above procedures were carried out with simple pipetting using a multi-well pipettor.

The DECODE protocol is designed to further increase throughput with automated

dispensers for almost all of its steps, and with a suitable automated dispenser, it is theoretically possible to complete 3 rounds of peptide selection for more than 96 antibody samples in less than 3 days with automation.

This information has been added on page 26, line 628.

*FastGene™ 96well PCR PLate Ultra Easy Cut (0.2mL/Non Skirted) (Nippon Genetics)

**Nunclon™ Sphera™ 96-Well, Nunclon Sphera-Treated, U-Shaped-Bottom Microplate (Thermo Scientific™)

concern #4

p.11, lines 248-254: What was the protein that C-10 was predicted to cross-react with in Fig. 3E, right (the DECODE score, 10982)? Have the authors confirmed by ELISA that C-10 actually binds to that protein? Is there any reason to believe that C-10 binding to that protein would affect the results of the immunostaining in Ref. 29?

Thank you very much for your valuable suggestions on cross-reactivity. In Fig. 3E, concerning the anti-c-fos antibody clone (C-10), we focused on four proteins (AHNAK2, LAMA1, FOSL2, PLCD4) that were predicted to have higher DECODE scores than the target protein (c-fos). We found that the regions on these proteins with the highest DECODE scores had sequences very similar to the C-10 motif. Particularly, FOSL2 belongs to the same FOS family as the c-fos protein, with very similar motifs and DECODE score values.

As additional experiments, we synthesized peptides from the regions on these four proteins with the highest DECODE scores and verified their binding reactions with anti-c-fos (C-10) by ELISA. We also confirmed the binding reaction of anti-c-fos (C-10) with the FOSL2 protein by ELISA. The results showed that anti-c-fos (C-10) exhibited cross-reactivity with antigen peptides derived from AHNAK2, LAMA1, and FOSL2, as well as the FOSL2 protein. The PLCD4-derived antigen peptide (pep4) was not tested for binding because it was hydrophobic and not soluble in buffer.

These findings indicate that in immunohistochemistry, especially of brain tissues, anti-c-fos (C-10) may stain not only the target protein c-fos but also LAMA1 or FOSL2. This poses a risk of misinterpretation in research results.

Thus, the ELISA results supported the reliability of the cross-reactivity predicted by the DECODE analysis and highlight the importance of predicting cross-reactivity in immunohistochemical research.

These results were added to Fig S3I and mentioned on line 262 of page 12.

concern #5

p.12: The results in Table S1 are very interesting. In particular, I have the following questions:

- For antibodies whose "species reactivity" includes not only human but also mouse and/or rat, are the motifs of the DECODE sequence in the antigen protein conserved in these species? Conversely, for human-specific antibodies, are there mutations in the DECODE site in mouse and rat?

We greatly appreciate your insightful comments on the interesting point about inter-species cross-reactivity. The 'species reactivity' listed in Table S1 refers to information provided by the antibody manufacturers on their websites.

Using the free multiple sequence alignment program Clustal Omega (https://www.ebi.ac.uk/jdispatcher/msa/clustalo), we aligned the target proteins of candidate species (human, mouse, rat) related to anti-c-fos and anti-p53 antibodies (attached below) to verify if the DECODE motifs are conserved across species.

The results indicated that for the anti-c-fos antibody, the DECODE sequence motifs were conserved among the species that the antibody manufacturer indicated as cross-reactive.

For the anti-p53 antibody (using antibodies from SantaCruz for peptide selection), the DECODE motifs were mostly conserved among the species that SantaCruz indicated as cross-reactive. However, significant mutations were observed between species for three specific anti-p53 antibodies (Pab1801, C-11, D-11).

For Pab1801, the DECODE motif matched the human sequence but showed mutations at the hotspot residues (D and W) in the sequences of mice and rats. Although SantaCruz indicates cross-reactivity with mice and rats, another supplier, Abcam, only lists reactivity with humans for the same clone. This indication by Abcam is consistent with the DECODE results.

Similarly, for Bp53-12, DO-1, and DO-7, SantaCruz lists cross-reactivity with human, mouse, and rat, but Abcam lists only human, specifically noting non-cross-reactivity with mouse and rat for DO-1, and this is also consistent with the DECODE results. Therefore, there is a risk that the species cross-reactivity provided by antibody manufacturers may be incorrect, necessitating caution.

On the other hand, for C-11 and D-11, which showed significant inter-species mutations, the ELISA results in Fig. 4D suggest that these antibodies have low binding affinity, making it difficult to discuss the conservation of DECODE motifs across species in the first place.

Additionally, it should be noted that even if an antibody manufacturer lists only one cross-reactive species, there could be conserved DECODE sequence motifs across other species. This absence of listing other species may simply indicate that the manufacturer did not test cross-reactivity, not necessarily that no cross-reactivity. Researchers also know from experience that the manufacturer's indication of cross reactivity is incorrect. In the present

crossreactivity results for several clones of p53, the consistency between Abcam's information and the DECODE results was high.

In conclusion, while the DECODE sequence motifs of antibodies showing cross-reactivity according to manufacturers are likely conserved among species, the cross-reactivity indicated by manufacturers may not always be accurate. To reliably predict inter-species cross-reactivity using the DECODE method, it is essential to analyze all species PDBs thoroughly.

This discussion has been added on page 20, line 466.

- Are there any characteristics specific to human epitopes that deviate from randomness in the DECODE peptide sequences in Table S1?

We are grateful for your very interesting comment. In this study, we did not discover epitopes specific to human proteins. It is possible that further large-scale epitope analysis in the future may reveal such characteristics.

- Were three rounds of selection performed for all antibodies in Table S1?

Thank you for your comment. For all antibodies in Table S1, we conducted three rounds of peptide selection. The sequence logos represent the motifs that converged by the third round. We mentioned this detail on line 282 of page 12 and added qPCR results for the cDNA-peptide complexes recovered at each round of peptide selection in Table S1. A smaller Ct value indicates greater peptide convergence. Using a fixed sequence mc1' specific to the anti-FLAG antibody as a positive control (see Table S2), we quantified the recovered cDNA-peptide, with a Ct value of around 7~9, using this as a reference for executing random sequence peptide selection.

- Are there any commonalities among the antibodies whose DECODE was "N.D"?

The antibodies that resulted in N.D. had highly random recovered peptides, which made it impossible to calculate the distance. Such results are caused by antibodies that recognize conformationally or modified amino acids, or by the loss of activity in binding condition.

Minor points

Thank you very much for the thorough review. We have revised concerns #6, 8-11 and indicated these with yellow markers. Additionally, regarding concern #7, we have added an explanation about BLOSUM62 on line 191 of page 9.

concern #6

p4, line 64: The K in "Kd" should be in italics.

p4. line 64.

concern #7

p8, line 184: It would be nice if there was a brief explanation of BLOSUM62 (using the example in Fig. 2A bottom).

We indicated that BLOSUM62 is an amino acid similarity scoring matrix used to compare a mino acid sequences on line 191 of page 9.

concern #8

p14, lines 320, 325, 332: The k in "kon" should be in italics.

concern #9

p25, line 596: The numbers in "H2SO4" should be subscripted.

concern #10

p26, line 629: The number in "CH2O" should be subscripted.

concern #11

pp.29-32: The journal names of references 6, 19, 23 and 33 should be abbreviated.

Thank you for pointing this out. All descriptions have been corrected.

Reviewer 2, Concern #5
Confirming the conservation of DECODE motifs among cross-species.

anti-c-fos antibodies

CLUSTAL O(1.2.4) MULTIPLE SEQUENCE ALIGNMENT

9F6 (CST: h,m,r)

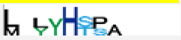

```
SP|P01100|FOS_HUMAN      MMFSGFNADYEASSSRCSSASPAGDSLSYYHSPADSFSSMGSPVNAQDFCTDLAVSSANF 60
SP|P01101|FOS_MOUSE      MMFSGFNADYEASSSRCSSASPAGDSLSYYHSPADSFSSMGSPVNTQDFCADLSVSSANF 60
SP|P12841|FOS_RAT        MMFSGFNADYEASSSRCSSASPAGDSLSYYHSPADSFSSMGSPVNTQDFCADLSVSSANF 60
                         *********************************************:****:**:******

SP|P01100|FOS_HUMAN      IPTVTAISTSPDLQWLVQPALVSSVAPSQTRAPHPFGVPAPSAGAYSRAGVVKTMTGGRA 120
SP|P01101|FOS_MOUSE      IPTVTAISTSPDLQWLVQPTLVSSVAPSQTRAPHPYGLPTQSAGAYARAGMVKTVSGGRA 120
SP|P12841|FOS_RAT        IPTVTAISTSPDLQWLVQPTLVSSVAPSQTRAPHPYGLPTPSTGAYARAGVVKTMSGGRA 120
                         ********[illegible]**:*:*: *:***:***:***::****
```

C-10 (SantaCruz: h, m, r)

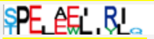

```
SP|P01100|FOS_HUMAN      QSIGRRGKVEQLSPEEEEKRRIRRERNKMAAAKCRNRRRELTDTLQAETDQLEDEKSALQ 180
SP|P01101|FOS_MOUSE      QSIGRRGKVEQLSPEEEEKRRIRRERNKMAAAKCRNRRRELTDTLQAETDQLEDEKSALQ 180
SP|P12841|FOS_RAT        QSIGRRGKVEQLSPEEEEKRRIRRERNKMAAAKCRNRRRELTDTLQAETDQLEDEKSALQ 180
                         ************************************************************

SP|P01100|FOS_HUMAN      TEIANLLKEKEKLEFILAAHRPACKIPDDLGFPEEMSVASLDLTGGLPEVATPESEEAFT 240
SP|P01101|FOS_MOUSE      TEIANLLKEKEKLEFILAAHRPACKIPDDLGFPEEMSVASLDLTGGLPEASTPESEEAFT 240
SP|P12841|FOS_RAT        TEIANLLKEKEKLEFILAAHRPACKIPNDLGFPEEMSVTSLDLTGGLPEATTPESEEAFT 240
                         ***************************:*[illegible]*.:*********
```

2H2 (abcam: h,m,r)

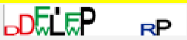

```
SP|P01100|FOS_HUMAN      LPLLNDPEPKPSVEPVKSISSMELKTEPFDDFLFPASSRPSGSETARSVPDMDLSGSFYA 300
SP|P01101|FOS_MOUSE      LPLLNDPEPKPSLEPVKSISNVELKAEPFDDFLFPASSRPSGSETSRSVPDVDLSGSFYA 300
SP|P12841|FOS_RAT        LPLLNDPEPKPSLEPVKNISNMELKAEPFDDFLFPASSRPSGSETARSVPDVDLSGSFYA 300
                         ************:****.**.:***:******************:*****:********

SP|P01100|FOS_HUMAN      ADWEPLHSGSLGMGPMATELEPLCTPVVTCTPSCTAYTSSFVFTYPEADSFPSCAAAHRK 360
SP|P01101|FOS_MOUSE      ADWEPLHSNSLGMGPMVTELEPLCTPVVTCTPGCTTYTSSFVFTYPEADSFPSCAAAHRK 360
SP|P12841|FOS_RAT        ADWEPLHSSSLGMGPMVTELEPLCTPVVTCTPSCTTYTSSFVFTYPEADSFPSCAAAHRK 360
                         ********.*******.***************.**:************************

SP|P01100|FOS_HUMAN      GSSSNEPSSDSLSSPTLLAL          380
SP|P01101|FOS_MOUSE      GSSSNEPSSDSLSSPTLLAL          380
SP|P12841|FOS_RAT        GSSSNEPSSDSLSSPTLLAL          380
                         ********************
```

anti-p53 antibodies

```
CLUSTAL O(1.2.4) MULTIPLE SEQUENCE ALIGNMENT
```

DO-2 (SantaCruz: h,m,r)

Bp53-12, DO1 (SantaCruz: h,m,r/Abcam: h) DO-7 (SantaCruz: h/Abcam: h)

Pab1801 (SantaCruz: h,m,r /Abcam: h)

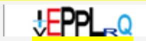
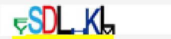
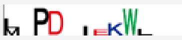

```
SP|P04637|P53_HUMAN      ---MEEPQSDPSVEPPLSQETFSDLWKLLPENNVLSPLP---SQAMDDLMLSPDDIEQWF 54
SP|P02340|P53_MOUSE      MTAMEESQSDISLELPLSQETFSGLWKLLPPEDILPS-----PHCMDDLLL-PQDVEEFF 54
SP|P10361|P53_RAT        ---MEDSQSDMSIELPLSQETFSCLWKLLPPDDILPTTATGSPNSMEDLFL-PQDVAELL 56
                                                *** :::*       :.*:**:* *:*: : :
```

C-11, D-11 (SantaCruz: h,m,r)

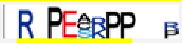

```
SP|P04637|P53_HUMAN      TEDPGPDEAPRMPEAAPPVAPAPAAPTPAAPAPAPSWPLSSSVPSQKTYQGSYGFRLGFL 114
SP|P02340|P53_MOUSE      E---GPSEALRVSGAPAAQDPVTETPGPVAPAPATPWPLSSFVPSQKTYQGNYGFHLGFL 111
SP|P10361|P53_RAT        E---GPEEALQVS-APAAQEPGTEAPAPVAPASATPWPLSSSVPSQKTYQGNYGFHLGFL 112
                          **.** ::  *     *   :* *.*** *  ***** *********.***:****

SP|P04637|P53_HUMAN      HSGTAKSVTCTYSPALNKMFCQLAKTCPVQLWVDSTPPPGTRVRAMAIYKQSQHMTEVVR 174
SP|P02340|P53_MOUSE      QSGTAKSVMCTYSPPLNKLFCQLAKTCPVQLWVSATPPAGSRVRAMAIYKKSQHMTEVVR 171
SP|P10361|P53_RAT        QSGTAKSVMCTYSISLNKLFCQLAKTCPVQLWVTSTPPPGTRVRAMAIYKKSQHMTEVVR 172
                      :******* ****  ***:****                                  **
```

Pab240 (SantaCruz: h,m,r/Abcam: h, m)

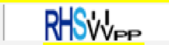

```
SP|P04637|P53_HUMAN      RCPHHERCSDSDGLAPPQHLIRVEGNLRVEYLDDRNTFRHSVVVPYEPPEVGSDCTTIHY 234
SP|P02340|P53_MOUSE      RCPHHERCSDGDGLAPPQHLIRVEGNLYPEYLEDRQTFRHSVVVPYEPPEAGSEYTTIHY 231
SP|P10361|P53_RAT        RCPHHERCSDGDGLAPPQHLIRVEGNPYAEYLDDRQTFRHSVVVPYEPPEVGSDYTTIHY 232
                      **********.***************   ***:**:**************.**: *****

SP|P04637|P53_HUMAN      NYMCNSSCMGGMNRRPILTIITLEDSSGNLLGRNSFEVRVCACPGRDRRTEEENLRKKGE 294
SP|P02340|P53_MOUSE      KYMCNSSCMGGMNRRPILTIITLEDSSGNLLGRDSFEVRVCACPGRDRRTEEENFRKKEV 291
SP|P10361|P53_RAT        KYMCNSSCMGGMNRRPILTIITLEDSSGNLLGRDSFEVRVCACPGRDRRTEEENFRKKEE 292
                      :********************************:********************:***

SP|P04637|P53_HUMAN      PHHELPPGSTKRALPNNTSSSPQPKKKPLDGEYFTLQIRGRERFEMFRELNEALELKDAQ 354
SP|P02340|P53_MOUSE      LCPELPPGSAKRALPTCTSASPPQKKKPLDGEYFTLKIRGRKRFEMFRELNEALELKDAH 351
SP|P10361|P53_RAT        HCPELPPGSAKRALPTSTSSSPQQKKKPLDGEYFTLKIRGRERFEMFRELNEALELKDAR 352
                            ******:*****. **:**  ************:****:*****************:

SP|P04637|P53_HUMAN      AGKEPGGSRAHSSHLKSKKGQSTSRHKKLMFKTEGPDSD     393
SP|P02340|P53_MOUSE      ATEESGDSRAHSSYLKTKKGQSTSRHKKTMVKKVGPDSD     390
SP|P10361|P53_RAT        AAEESGDSRAHSSYPKTKKGQSTSRHKKPMIKKVGPDSD     391
                      * :* *.******: *:*********** *.*. *****
```
